# Orbitofrontal-striatal potentiation underlies cocaine-induced hyperactivity

**DOI:** 10.1038/s41467-020-17763-8

**Published:** 2020-08-10

**Authors:** Sebastiano Bariselli, Nanami L. Miyazaki, Meaghan C. Creed, Alexxai V. Kravitz

**Affiliations:** 1 National Institute of Diabetes and Digestive and Kidney Diseases, National Institutes of Health, Bethesda, MD 20892 USA; 2 Washington University Pain Center, St Louis, MO 63110 USA; 3 Departments of Psychiatry, Anesthesiology, and Neuroscience, Washington University School of Medicine, St Louis, MO 63110 USA; 4 Present Address: National Institute on Alcohol Abuse and Alcoholism (NIAAA), Laboratory for Integrative Neuroscience (LIN), Bethesda, MD 20892-9412 USA

**Keywords:** Neuroscience, Motor control, Basal ganglia

## Abstract

Psychomotor stimulants increase dopamine levels in the striatum and promote locomotion; however, their effects on striatal pathway function in vivo remain unclear. One model that has been proposed to account for these motor effects suggests that stimulants drive hyperactivity via activation and inhibition of direct and indirect pathway striatal neurons, respectively. Although this hypothesis is consistent with the cellular actions of dopamine receptors and received support from optogenetic and chemogenetic studies, it has been rarely tested with in vivo recordings. Here, we test this model and observe that cocaine increases the activity of both pathways in the striatum of awake mice. These changes are linked to a dopamine-dependent cocaine-induced strengthening of upstream orbitofrontal cortex (OFC) inputs to the dorsomedial striatum (DMS) in vivo. Finally, depressing OFC-DMS pathway with a high frequency stimulation protocol in awake mice over-powers the cocaine-induced potentiation of OFC-DMS pathway and attenuates the expression of locomotor sensitization, directly linking OFC-DMS potentiation to cocaine-induced hyperactivity.

## Abstract (web summary)

Psychomotor stimulants increase dopamine levels in the striatum and promote locomotion but their effects on striatal pathways in vivo remain unclear. The authors show that cocaine increases the activity of direct and indirect pathway striatal neurons of awake mice via the orbitofrontal cortex.

## Introduction

Exposure to psychomotor stimulants induces a plethora of neurobehavioral effects that range from hyperactivity, cognitive impairments, sensitization, and tolerance, which can eventually lead to substance abuse^1^. However, despite being one of the most widely reported effects of cocaine and amphetamine, it is not clear why stimulants cause hyperactivity. Psychostimulants increase dopamine levels in striatal regions^2^, which is theorized to increase and decrease the activity of direct (dMSNs) and indirect (iMSNs) medium spiny neurons, via actions on dopamine 1 (D1R)-like and D2-like receptors^3–5^, respectively. Optogenetic^6–8^, chemogenetic^9–11^, synaptic inhibition^12^ and in vivo calcium-activity^13–15^ studies support a model by which bidirectional modulation of these striatal pathways controls behavioral responses to psychostimulants. Moreover, recent work indicates that lateral inhibition between dMSNs and iMSNs plays a pivotal role in determining the locomotor effects of cocaine^16,17^, which is consistent with the idea that opposing activation in each pathway underlies cocaine-induced hyperactivity. While this model links cellular actions of cocaine to dopamine receptor function, there have been surprisingly few direct tests of the hypothesis that cocaine excites direct pathway neurons and inhibits indirect pathway neurons in awake animals.

Studies that utilized immediate early genes (IEGs) as a proxy for neural activity report that phospho-Erk, MSK1, and phospho-histone H3 were activated selectively in direct pathway neurons, while other IEGs such as c-Fos and zif268 were activated in both pathways^18^. Another study reported that cocaine enhanced c-Fos expression in direct pathway neurons but had no observable effect in indirect pathway neurons^19^. However, IEGs are expressed at low basal levels in striatum so this approach is not ideal for testing whether cocaine also inhibits indirect pathway neurons in vivo. To our knowledge, only one study has included cell-type specific calcium recordings of the dorsal striatum of awake mice, and reported that, on average, cocaine inhibited both pathways^20^. Here, we expand on this point using population calcium recording of each pathway during exposure to cocaine and found that, in contrast to the previously calcium imaging result but consistent with reported IEG activation, cocaine increased the frequency of calcium events in both populations.

To explain why both pathways would have heightened activity, we hypothesized that cocaine may strengthen excitatory inputs to the striatum. Dopamine, psychostimulants and other drugs of abuse can modulate synaptic transmission from cortical inputs^21,22^, and this can control striatal activity^23^. The OFC projection to the striatum is a particularly important input for multiple effects of stimulants. Hyperlocomotion and sensitization^24^, place preference^25^, cue-induced reinstatement^26,27^, compulsive behavior^28^ and cognitive dysfunctions^29,30^ have been linked to abnormalities in orbitofrontal cortex (OFC)-striatal circuits following cocaine exposure and withdrawal or dopamine neuron self-stimulation. Ex vivo studies have also linked a potentiation of OFC inputs onto dorsomedial striatum (DMS) neurons to some of these behavioral maladaptations^24,28^. Therefore, we developed a model for examining OFC-DMS connectivity in vivo in awake mice to test our hypothesis that cocaine potentiates OFC-DMS inputs. Briefly, we expressed an excitatory opsin in the OFC and implanted recording wires in the downstream DMS. We delivered short “test pulses” to the OFC and measured the resulting evoked LFPs in the DMS as animals were exposed to cocaine and other stimulants. Using this model, we found that cocaine causes a dopamine-dependent potentiation of OFC-DMS inputs. Two other stimulants recapitulated this increase in OFC-DMS connectivity, which was blocked by pre-treatment with dopaminergic antagonists. Further, the enhanced OFC-DMS pathway function was associated with an increased OFC-evoked firing in striatal neurons, indicating a concomitant increase of OFC input efficacy. Together, our results support our hypothesis, demonstrating that psychostimulants potentiate OFC light-evoked responses in the DMS, which enhance OFC control over DMS output.

To test the necessity of OFC-DMS potentiation for cocaine-induced hyperactivity, we asked whether we could de-potentiate OFC-DMS inputs to counteract the increased striatal neural activity, and thus attenuate cocaine-induced hyperlocomotion. Cortico-striatal pathway stimulation can alleviate behavioral symptoms of neurological and psychiatric disorders^31^, including drug-related behavior in rodents^32–34^ and humans^35^. In particular, clinical and pre-clinical^36^ results show that high-frequency deep brain stimulation (DBS) of different ventral striatum sub-regions attenuates drug-seeking behavior^37^. In addition, 1 Hz and 12 Hz optogenetic or electrical stimulation of prefrontal cortex (PFC) inputs induce long-term depression (LTD) in nucleus accumbens (NAc) slices and attenuate cocaine-seeking behavior^32^ and locomotor sensitization in rodents^33,34^, respectively. Cortical inputs onto DMS can also undergo plasticity: while low-frequency^38^ and high-frequency stimulation^39^ promote LTD, theta-burst stimulation (TBS)^40^ induces long-term potentiation (LTP) ex vivo. Importantly, low-frequency stimulation of OFC inputs combined with a D1R-antagonist induces LTD in dorsal striatum brain slices, and attenuates behavioral perseveration in compulsive mice^28^. However, all prior work on this topic was done in the ex vivo slice preparation, and the effects of stimulation protocols on OFC-DMS circuit function of awake animals have not been examined. To address this, we tested the ability of multiple plasticity protocols to modify the strength of the OFC-DMS projection, in awake mice, while monitoring evoked responses in this circuit in real-time. We identified a high-frequency stimulation protocol that robustly depressed the OFC-DMS evoked responses, and found that this same protocol also attenuated the cocaine-induced OFC-DMS potentiation and the hyper-locomotor actions of cocaine in vivo. Altogether, these data suggest that cocaine facilitates OFC-DMS synapses, increasing both direct and indirect pathway activity in the striatum of awake mice, and thereby driving cocaine-induced hyperactivity.

## Results

### Cocaine exposure increases neuronal activity in dorsomedial striatum in vivo

**Fig. 1:**
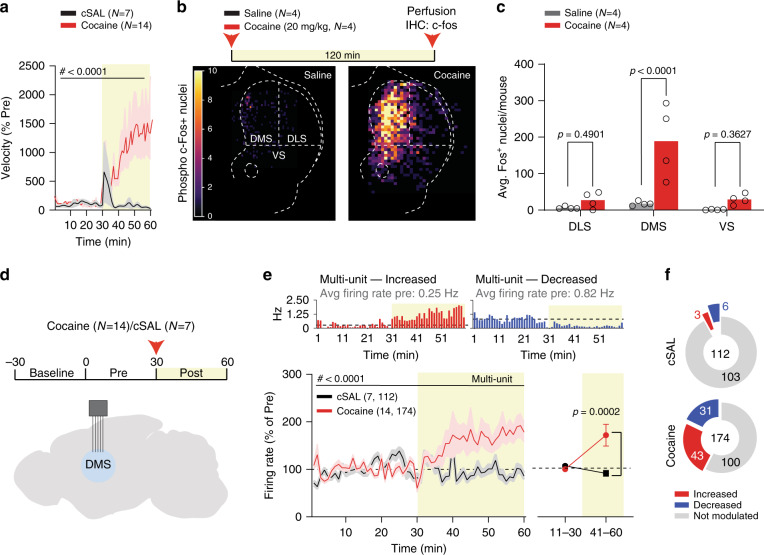
Cocaine increases neuronal activity in dorsomedial striatum. **a** Norm. velocity for saline (cSAL) and cocaine (RM two-way ANOVA; time main effect: *F*_(59,1121)_  = 2.765, *p* < 0.0001; drug main effect: *F*_(1,19)_ = 3.829, *p* = 0.0652; time Χ drug interaction: *F*_(59,1121)_ = 3.044, *p* < 0.0001). **b** Experimental paradigm and heat-map of c-fos expression after i.p. saline or cocaine injections. **c** Averaged per-mouse phospho-c-fos positive nuclei in dorsolateral (DLS), dorsomedial (DMS) and ventral striatum (VS; RM two-way ANOVA; region main effect: *F*_(2,12)_ = 13.84, *p* = 0.0008; drug main effect: *F*_(1,6)_  = 13.18, *p* = 0.0110; region Χ drug interaction: *F*_(2,12)_ = 9.789, *p* = 0.0030; followed by between-subject post-hoc False Discovery Rate method of Benjamini, Krieger and Yekuteli test for saline vs cocaine; DLS *t*_(18)_ = 0.7046; DMS *t*_(18)_ = 5.712; VS *t*_(18)_ =  0.9339). **d** Schematics of brain implants and experimental time-course. **e** Time-course of normalized multi-unit activity (RM two-way ANOVA; time main effect: *F*_(3.063,869.8)_ = 3.118, *p* = 0.0246; drug main effect: *F*_(1,284)_ = 6.076, *p* = 0.0143, time Χ drug interaction: *F*_(59,16756)_ = 4.180, *p* < 0.0001) and binned analysis of normalized multi-unit activity (RM two-way ANOVA; time main effect: *F*_(1,284)_ = 3.732, *p* = 0.0544; drug main effect: *F*_(1,284)_  = 6.511, *p* = 0.0112; time Χ drug interaction: *F*_(1,284)_ = 9.185, *p* = 0.0027, followed by between-subject Bonferroni post hoc test cSAL vs COC; 11–30: *t*_(568)_ = 0.3359, *p* > 0.9999; 41–60: *t*_(568.0)_ = 3.947). Insets at top: example rate histograms showing multi-units that increased and decreased firing after cocaine. **f** Pie-charts of non-modulated, potentiated or depressed multi-units after saline or cocaine i.p. injections. Data are represented as single points and/or as mean ± SEM (standard error of the mean). *N* and *n* indicate number of mice and multi-units, respectively.

To investigate the effects of cocaine on striatal circuits, animals received an intraperitoneal (i.p.) injection of cocaine (20 mg/Kg) or saline (cSAL) as control. Cocaine strongly increased locomotor activity (Fig. 1a) and the expression of phospho c-Fos, a marker of neuronal activation in the striatum (Fig. 1b, c). More specifically, cocaine significantly increased average c-Fos expression in the dorsomedial portion of the striatum (DMS) compared to saline, with non-significant increases in dorsolateral (DLS) and ventral striatum (VS; Fig. 1c). To investigate whether the increased c-Fos expression was associated with an increased neuronal firing, we implanted 32-channel arrays in DMS for in vivo electrophysiology recordings. Animals were placed in an open-field and neuronal activity was recorded for 30 minutes before and after an i.p. injection of either saline (cSAL, *n* = 7 mice) or cocaine (*n* = 14 mice, Fig. 1d). We recorded a total of 112 and 174 multi-units from saline and cocaine exposed animals, respectively. Ten minutes post injection, cocaine increased both the firing rate (Fig. 1e) and the number of modulated units (Fig. 1f and Supplementary Fig. 1a) compared to saline. In addition, cocaine increased gamma power in DMS (Supplementary Fig. 1b, c), which has been linked to BOLD signals in humans^41^. Altogether, these data demonstrate that cocaine increases neuronal activity in the DMS.

### Cocaine exposure increases calcium population activity in both direct and indirect pathway

**Fig. 2:**
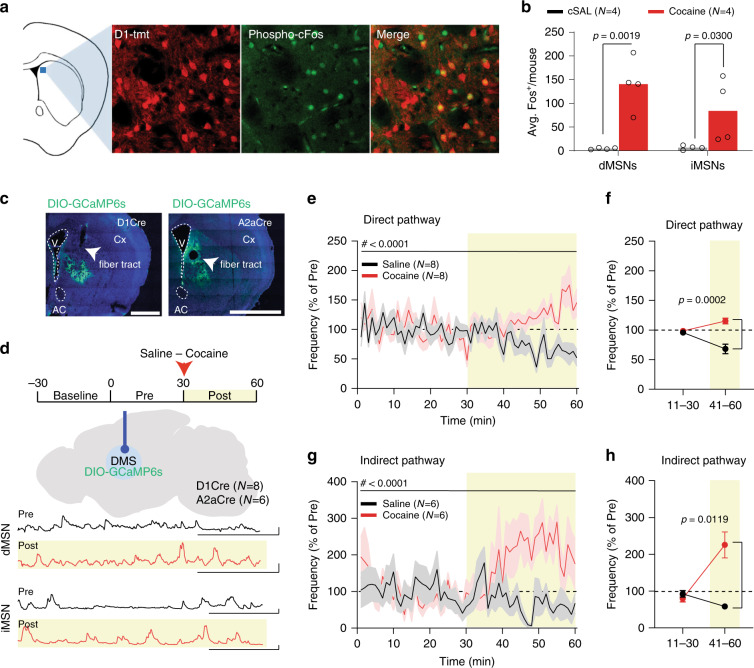
Cocaine increases population activity of both direct and indirect pathway in DMS. **a** Representative images of phospho-c-fos staining: D1-tmt (D1-tomato; red) and phospho-c-fos (green). Image area is a 200 µm square of tissue. **b** Averaged per-mouse (*n* = 4 mice per condition) c-fos expression after i.p. saline (cSAL) or cocaine injections in dMSNs and iMSNs (RM two-way ANOVA; cell-type main effect: *F*_(1,6)_ = 3.998, *p* = 0.0925; drug main effect: *F*_(1,6)_ = 14.53, *p* = 0.0088; cell-type Χ drug interaction: *F*_(1,6)_ = 4.634, *p* = 0.0748; followed by between-subject post hoc False Discovery Rate method of Benjamini, Krieger and Yekuteli test saline vs cocaine; dMSNs: *t*_(12)_ = 4.370; iMSN: *t*_(12)_ = 2.488). **c** Representative images of GCaMP6s expression in DMS (V ventricle, AC anterior commissure, Cx cortex). Scale bar: 1 mm. **d** Experimental and brain schematics of fiber photometry experiments with example photometry traces. Scale bar: 30 ms, 5% dF/F. **e** Norm. frequency of direct pathway calcium events (RM two-way ANOVA by both factors; time main effect: *F*_(59,413)_ = 1.063, *p* = 0.3585; drug main effect: *F*_(1,7)_ = 20.37, *p* = 0.0028; time Χ drug interaction: *F*_(59,413)_ = 2.891, *p* < 0.0001). **f** Binned norm. frequency (RM two-way ANOVA by both factors; time main effect: *F*_(1,7)_ =  0.9040, *p* = 0.3734; drug main effect: *F*_(1,7)_ = 53.77, *p* = 0.0002, time Χ drug interaction: *F*_(1,7)_ = 72.32, *p* < 0.0001, followed by between-subject Bonferroni post-hoc test Saline vs Cocaine; 11–30: *t*_(7)_ = 1.539, *p* = 0.3354; 31–60: *t*_(7)_ = 8.137). **g** Norm. frequency of indirect pathway calcium events (RM two-way ANOVA by both factors; time main effect: *F*_(59,295)_ = 1.118, *p* = 0.2729; drug main effect: *F*_(1,5)_ = 21.45, *p* = 0.0057; time Χ drug interaction: *F*_(59,295)_ = 2.5, *p* < 0.0001). **h** Binned frequency (RM two-way ANOVA by both factors; time main effect: *F*_(1,5)_ = 9.215, *p* = 0.0289; drug main effect: *F*_(1,5)_ = 13.48, *p* = 0.0144; time Χ drug interaction: *F*_(1,5)_ = 25.37, *p* = 0.0040; followed by between-subject Bonferroni post-hoc test Saline vs Cocaine; 11–30: *t*_(5)_ = 0.9309, *p* = 0.7893; 31–60: *t*_(5)_ = 4.583). Data are represented as single points and/or as mean ± SEM. *N* indicates number of mice.

Interestingly, a population of multi-units in our recordings was inhibited by cocaine (Fig. 1f and Supplementary Fig. 1a), suggesting that cocaine exerts bidirectional actions on different groups of neurons. To investigate whether the bidirectional changes in multi-unit firing corresponded to a, respectively, increased and decreased activity of direct and indirect pathway, we analysed the expression of phospho-c-Fos in dMSNs or iMSNs, differentiated by genetic expression of a fluorescent label. A by-mouse analysis revealed that cocaine increased c-fos expression in both dMSNs (*n* = 4 mice) and iMSNs (*n* = 4 mice; Fig. 2a, b). To follow up on this with a real-time assessment of how cocaine modulates direct and indirect pathway, D1Cre (*n* = 8) and A2aCre (*n* = 6) mice were transduced with a Cre-dependent DIO-GCaMP6s virus (Fig. 2c), while WT (*n* = 8) mice were transduced with a virus expressing eYFP. Mice were implanted with an optic fiber in the DMS to record bulk calcium signal from each pathway in this region (Fig. 2d). We used the eYFP animals to characterize potential movement artifacts in our recordings and found that 7/8 eYFP animals did not exhibit any local maxima in the fluorescence signal that exceeded 5% dF/F (Supplementary Fig. 2a). We therefore used this as a cut-off for potential movement artifacts and limited our analyses of calcium transients to those that were above 5% dF/F (Supplementary Fig. 2b). D1Cre and A2aCre mice had similar frequencies of calcium transients during pre-cocaine period (Supplementary Fig. 2b) and comparable hyper-locomotor responses to cocaine (Supplementary Fig. 2c). Surprisingly, cocaine increased population transient frequency in both direct (Fig. 2e, f) and indirect pathway (Fig. 2g, h) compared to saline controls. All mice (*n* = 6 iMSN, *n* = 8 dMSN mice) increased velocity with cocaine, and 12/14 mice had an increase in calcium transient frequency after cocaine. However, these two factors did not themselves correlate, demonstrating that the increase in calcium frequency was not solely attributable to the increase in velocity (Supplementary Fig. 2d). Together, our c-Fos and in vivo photometry data indicate that cocaine increases calcium activity in direct and indirect striatal pathway neurons of the DMS.

### Cocaine exposure potentiates OFC-DMS inputs

**Fig. 3:**
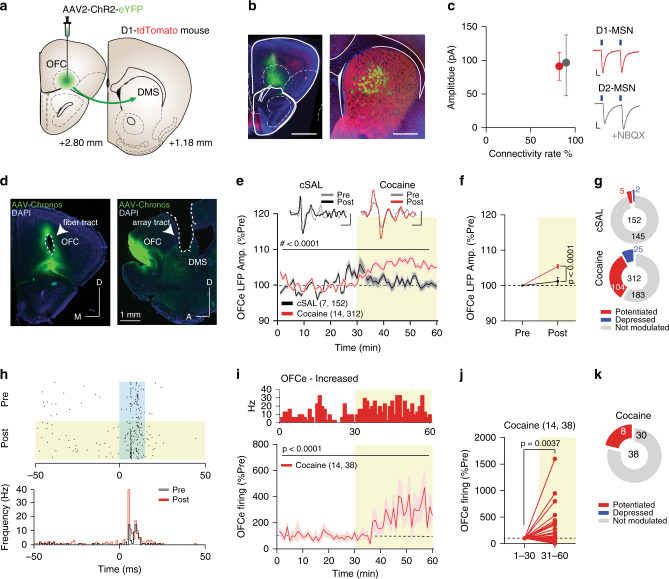
Cocaine increases OFC-DMS pathway efficacy. **a** Schematic of ex vivo recordings. ChR2-eYFP was targeted to the OFC and slice recordings were performed in the DMS of D1-tmt mice. **b** Example histology showing fluorescence in OFC and fibers in DMS. Scale bar: 1 mm. **c** Connectivity rates between OFC and dMSNs or putative iMSNs. Right: example currents evoked by 4 ms illumination with blue light. Scale bar: 10 ms, 20 pA. **d** Representative images of Chronos expression in OFC with fibers projecting to DMS. **e** Time-course of normalized OFCe LFP amplitude for cocaine and saline (cSAL) (two-way ANOVA; time main effect: *F*_(59,27720)_ = 13.18, *p* < 0.0001; drug main effect: *F*_(1,27720)_ = 225.7, *p* < 0.0001; time Χ drug interaction: *F*_(59, 27720)_ = 10.27, *p* < 0.0001). Scale bar: 20 ms, 2 μV. **f** Binned OFCe LFP amplitude (two-way ANOVA; time main effect: *F*_(1,924)_ = 35.11, *p* < 0.0001; drug main effect: *F*_(1,924)_ = 14.27, *p* = 0.0002, time Χ drug interaction: *F*_(1,924)_ = 14.27, *p* = 0.0002; followed by between-subject Bonferroni post hoc test; Pre: cSAL vs cocaine: *t*_(924)_  = 0, *p* > 0.9999; post: *t*_(924)_ = 5.342). **g** Pie-charts reporting the number of significantly potentiated, depressed or non-significantly modulated OFCe LFP responses. **h** Representative raster-plot and peri-stimulation histogram of OFCe responses. **i** Time-course of normalized OFC-stimulation evoked firing rate (RM one-way ANOVA, *F*_(59,2183)_ = 3.056). **j** Binned OFCe firing frequency in response to cocaine (two-tailed Wilcoxon matched pairs signed rank test: *W* = 392.0). **k** Pie-charts reporting the number of OFCe firing responses. Data are represented as single points and/or as mean ± SEM. *N*, *n* indicate number of mice and OFCe LFPs or units included in the analysis.

It is difficult to reconcile the cocaine-mediated increase in indirect pathway activity with the inhibitory actions of D2R activation on iMSN function^18,21,42,43^. To account for why indirect pathway neurons were more active when the mouse was exposed to cocaine, we hypothesized that cocaine may increase glutamatergic drive onto both iMSNs and dMSNs. Specifically, we hypothesized that cocaine may potentiate OFC-DMS pathway, either by enhancing synaptic connections^28^ or promoting “up-states” in striatal neurons^23^. We first investigated whether OFC neurons preferentially innervate either direct or indirect striatal neurons. To test this, we expressed ChR2 in the OFC of D1-tomato (D1-tmt) mice^44^ and patched D1tmt-positive or D1tmt-negative neurons in the DMS to identify dMSNs and putative iMSNs. We tested each patched neuron for connectivity and measured the amplitude of the evoked current. Similar to previous reports^24,28^, we found that the OFC projects to both dMSNs and iMSNs, with no observed differences in connectivity rate or strength (Fig. 3a–c).

To test whether cocaine potentiated OFC-DMS inputs in vivo, we expressed the blue light-activated opsin Chronos^45^ in the OFC (Fig. 3d) and measured light-evoked changes in striatal local field potentials (LFPs), a measure of cortical-input efficacy^46,47^ before (Pre) and after (Post) mice received an i.p. injection of cocaine or saline (Cocaine, *n* = 14 mice, and cSAL, *n* = 7 mice, Fig. 3d). We included wires with evoked LFP responses for further analysis (see Methods). This included 312 wires for cocaine, and 152 wires for saline. As we hypothesized, cocaine increased the amplitude of OFC-evoked (OFCe) striatal LFP responses (Fig. 3e–g). This effect was also observed at a per-mouse level, when OFCe LFP responses from each mouse were averaged (Supplementary Fig. 3a). Next, to investigate whether changes in evoked LFPs were associated with alterations in neuronal firing, we examined whether cocaine also potentiated OFCe spiking in the striatum. Thirty-eight of 197 (22%) recorded striatal multi-units were activated by OFC stimulation, consistent with a glutamatergic input from OFC (Supplementary Fig. 3b–d). Similar to the effect we observed on OFCe LFPs, cocaine also potentiated OFCe spiking in these units (Fig. 3h–k). Thus, we conclude that cocaine increases the synaptic efficacy of OFC inputs onto striatal neurons, which may be the source of enhanced spiking in both pathways during cocaine exposure.

### Cocaine-mediated OFC-DMS potentiation is dopamine-dependent

**Fig. 4:**
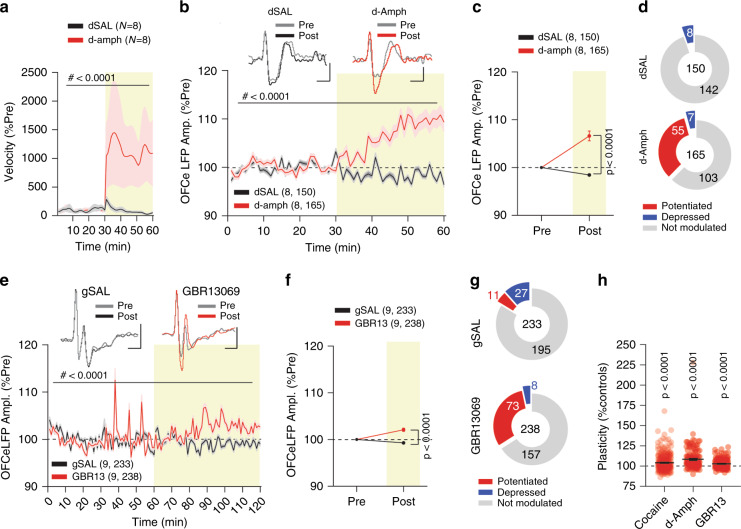
Dopamine-dependent OFC-DMS input strengthening. **a** Time-course of norm. velocity for saline (dSAL) and d-amphetamine (d-amph) (RM two-way ANOVA, time main effect: *F*_(1.064,14.90)_ = 2.685, *p* = 0.1209; drug main effect: *F*_(1,14)_  = 3.039, *p* = 0.1032; time Χ drug interaction: *F*_(59,826)_ = 2.753, *p* < 0.0001). **b** Time-course of normalized OFCe LFP responses (two-way ANOVA; time main effect: *F*_(59,18780)_  = 10.90, *p* < 0.0001; drug main effect: *F*_(1,18780)_ = 744.80, *p* < 0.0001; time Χ drug interaction: *F*_(59,18780)_ = 18.63, *p* < 0.0001). Scale bar: 20 ms, 2 μV. **c** Binned OFCe LFP amplitude (two-way ANOVA; time main effect: *F*_(1,626)_ = 21.20, *p* < 0.0001; drug main effect: *F*_(1,626)_ = 54.76, *p* < 0.0001; time Χ drug interaction: *F*_(1,626)_ = 54.76, *p* < 0.0001, followed by between-subject Bonferroni post-hoc test dSAL vs d-Amph; 1–30: *t*_(626)_  = 0, *p* > 0.9999; 31–60: *t*_(626)_ = 10.47). **d** Pie-charts with numbers of significantly potentiated or depressed and not modulated OFCe LFP responses. **e** Time-course of normalized OFCe LFP responses for saline (gSAL) and GBR13069 (GBR13) (RM two-way ANOVA; time main effect: *F*_(119,56280)_ = 13.83, *p* < 0.0001; drug main effect: *F*_(1,56280)_ = 415.1, *p* < 0.0001; time Χ drug interaction: *F*_(119,56280)_ = 16.61, *p* < 0.0001). Scale bar: 20 ms, 2 μV. **f** Binned OFCe LFP amplitude (two-way ANOVA; time main effect: *F*_(1,938)_ = 12.42, *p* = 0.0004; drug main effect: *F*_(1,938)_ = 50.17, *p* < 0.0001; time Χ drug interaction: *F*_(1,938)_ = 50.17, *p* < 0.0001; followed by between-subject Bonferroni post-hoc test gSAL vs GBR13; 1–30: *t*_(938)_ = 0, *p* > 0.9999; 31–60: *t*_(938)_ = 10.02). **g** Pie-charts reporting the number of significantly potentiated or depressed and not modulated OFCe LFP responses. **h** Percentage change in OFCe LFPs (one-way ANOVA, *F*_(2,712)_ = 12.76, *p* < 0.0001, followed by one-sided *t*-test to 100%, cocaine: *t*_(311)_ = 9.469; d-amph: *t*_(164)_ = 6.421, GBR13: *t*_(237)_ = 6.120). Data are represented as single points and/or as mean ± SEM. N,n indicate number of mice and OFCe LFPs included in the analysis.

**Fig. 5:**
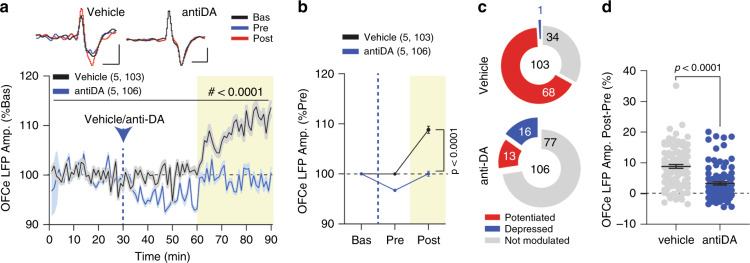
Dopaminergic antagonists prevent cocaine-induced OFC-DMS potentiation. **a** Time-course of normalized OFCe LFP amplitude expressed as a percentage of baseline prior to vehicle or anti-DA i.p. injections (RM two-way ANOVA; time main effect *F*_(3.568,738.7)_ = 19.79, *p* < 0.0001, drug main effect: *F*_(1,107)_ =  133.8, *p* < 0.0001, time Χ drug interaction: *F*_(89,18423)_ = 14.18, *p* < 0.0001). Scale bar: 20 ms, 5 μV. **b** Binned OFCe LFP responses (RM two-way ANOVA, time main effect: *F*_(1.277,264.3)_ = 150.4, *p* < 0.0001; drug main effect: *F*_(1,207)_ = 122.3, *p* < 0.0001; time Χ drug: *F*_(2,414)_ = 75.39, *p* < 0.0001; followed by between-subject Bonferroni post hoc test antiDA vs Veh; 1–30: *t*_(621)_ = 0, *p* > 0.9999; 31–60: *t*_(621)_ = 5.923; 61–90: *t*_(621)_ = 15.87). **c** Pie-charts reporting the number of significantly potentiated or depressed and not modulated OFCe LFP responses. **d** Change in OFCe LFP amplitude (Post - Pre) expressed as percentage of baseline (two-tailed Mann–Whitney U = 2627). Data are represented as single points and/or as mean ± SEM. *N*, *n* indicate number of mice and OFCe LFPs included in the analysis.

To investigate whether OFC-DMS potentiation was dependent on dopamine, we first tested whether this potentiation occurred with other psychostimulants. Mice that were implanted with 32-channel arrays in DMS were i.p. injected with d-amphetamine (d-amph, *n* = 8 mice) or saline as controls (dSAL, *n* = 8 mice). We observed that at the dose of 3 mg/Kg, d-amphetamine increased locomotor activity (Fig. 4a), OFCe LFP amplitudes (Fig. 4b, c), and the number of potentiated LFPs (Fig. 4d) compared to saline. As with cocaine, a between-subject analysis against saline revealed that d-amph increased averaged responses per mouse as well (Supplementary Fig. 4a). Considering that cocaine and amphetamine both have multiple mechanisms of action^48^, we sought to selectively isolate the contribution of dopamine to the potentiation of OFC-DMS connection strength. To do this, we assessed whether an i.p. injection of a selective dopamine transporter (DAT) blocker^49^, GBR13069, recapitulated the effects induced by cocaine and amphetamine. Although not as robust as cocaine and amphetamine, GBR13069 (*n* = 9 mice) significantly increased both locomotor activity (Supplementary Fig. 4b) and OFCe LFP responses (Fig. 4e–h) compared to saline controls (gSAL, *n* = 9 mice). Finally, we blocked dopamine receptors to test the necessity of dopamine for potentiating OFC-DMS connection. Mice received either an i.p. injection of a mixture of antagonists for D1R (SCH23390; 0.15 mg/Kg) and D2R (sulpiride; 25 mg/Kg; Fig. 5a), named anti-DA (*n* = 5 mice), or vehicle as control (*n* = 5 mice). Anti-DA injection caused a slight depression in OFCe LFP amplitude when normalized to baseline (Fig. 5a), indicating that physiological dopamine receptor activation controls OFC-DMS pathway input strength under basal conditions. Compared to vehicle, pre-treatment with anti-DA also blunted both the cocaine-induced hyperlocomotion (Supplementary Fig. 4c) and the increase of OFCe LFP amplitude (Fig. 5a–c), even when normalizing for the anti-DA mediated reduction in OFCe LFPs (Fig. 5d). Finally, we injected these mice with cocaine after pre-treating them with either vehicle or a mixture of a lower dose of SCH23390 (0.03 mg/Kg) and the same dose of sulpiride (25 mg/Kg), named “low anti-DA”. Analysing the same wires as with the higher dose, we found that reducing the dose of D1R antagonist was permissive for OFCe LFP potentiation (Supplementary Fig. 4d), as no differences were detected compared to vehicle. Thus, we conclude that psychostimulant-induced increase in OFC-DMS input strength depends on dopamine and can be blocked by a D1-antagonist in a dose-dependent manner in awake mice.

### Optogenetic-mediated high-frequency stimulation depresses OFC-DMS pathway and locomotion

**Fig. 6:**
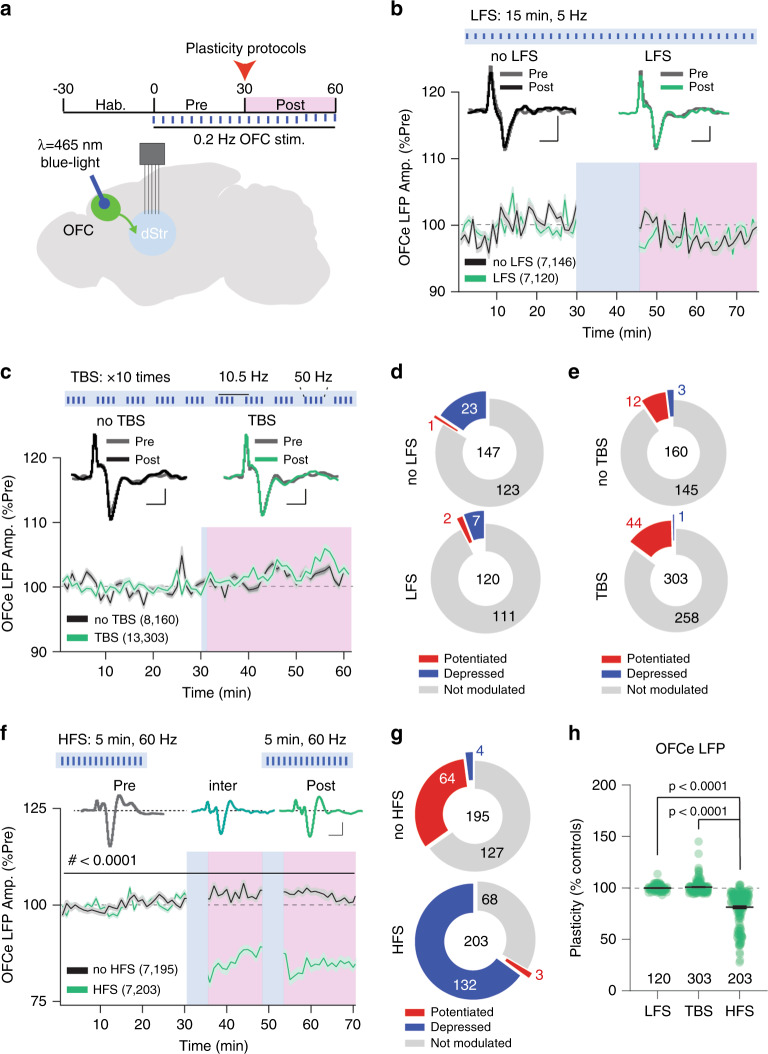
HFS induces long-lasting depression at OFC-DMS pathway. **a** Experimental paradigm and brain schematic for optogenetic stimulation experiments. **b** Time-course of normalized OFCe LFP response amplitude upon low-frequency stimulation (LFS) or no LFS (RM two-way ANOVA; time main effect: *F*_(18.83,4970)_ = 13.24, *p* < 0.0001; protocol main effect: *F*_(1,264)_ = 0.007208, p = 0.9324; time Χ protocol interaction: *F*_(59,15576)_ = 6.597, *p* < 0.0001). Scale bar: 20 ms, 5 μV. **c** Time-course of normalized OFCe LFP response amplitude upon theta-burst stimulation (TBS) or no TBS (RM two-way ANOVA; time main effect: *F*_(59,27660)_ = 13.50, *p* < 0.0001, protocol main effect: *F*_(1,27660)_ = 20.58, *p* < 0.0001; time Χ protocol interaction: *F*_(59,27660)_ = 5.518, *p* < 0.0001). Scale bar: 20 ms, 5 μV. **d** Pie-charts reporting the number of significantly potentiated or depressed and non-modulated OFCe LFP responses upon LFS or no LFS. **e** Pie-charts reporting the number of significantly potentiated or depressed and non-modulated OFCe LFP responses upon TBS or no TBS. **f** Time-course of norm. OFCe LFP upon high-frequency stimulation (HFS) or no HFS (RM two-way ANOVA; time main effect: *F*_(73,28908)_ = 39.19, *p* < 0.0001; protocol main effect: *F*_(1,396)_ = 205.2, *p* < 0.0001; time Χ protocol interaction: *F*_(73,28908)_ = 89.20, *p* < 0.0001). Scale bar: 20 ms, 5 μV. **g** Pie-charts reporting the number of significantly potentiated or depressed and non-modulated OFCe LFP responses. **h** Scatter-plot reporting the change in OFCe LFP responses relative to controls for LFS, TBS and HFS (one-way ANOVA; *F*_(3,774)_ = 181.7, *p* < 0.0001 followed by between-subject Dunnett’s test; HFS vs LFS: *q*_(774)_ = 16.72; HFS vs TBS: *q*_(774)_ = 22.25; HFS vs sHFS: *q*_(774)_ = 12.42). Data are represented as mean ±  SEM. *N*, *n* indicate number of mice and OFCe LFPs included in the analysis.

As cocaine potentiated OFC-DMS inputs, we asked whether targeted, plasticity-inducing stimulation protocols could depress this input and block cocaine-induced behaviour, building on similar approaches used by other groups^32,34,35,50^. We tested the effect of multiple stimulation protocols in vivo, based on those that have been shown to induce plasticity ex vivo (Fig. 6a). To monitor circuit plasticity as it occurs in vivo, we investigated the consequences of low-frequency (LFS: 5 Hz for 15 min; *n* = 7 mice for noLFS and *n* = 7 mice for LFS) and theta-burst (TBS: 10 repetitions of 40 stimulations organized in trains of 50 Hz every 10.5 Hz; *n* = 8 mice for noTBS and *n* = 13 mice for TBS) stimulation of OFC on the amplitude of OFCe LFP responses in DMS. Surprisingly, both LFS and TBS failed to induce an observable plasticity in our awake preparation (Fig. 6b–e). Next, we sought to explore the effects of HFS at OFC-DMS inputs, which induces LTD at cortico-striatal inputs in slice preparations^39^ and anesthetized animals^51^. Because of constraints related to Chronos-induced spike fidelity^45^, we limited our stimulation to 60 Hz. Moreover, to understand whether HFS had any short-/long-term or additive effects^52^, we delivered two 5-min 60 Hz periods (ON) interleaved by a 14-min pause (interHFS) and followed by an OFF period (post HFS). Compared to noHFS control, HFS strongly depressed OFCe LFP responses (during the inter-HFS phase, Fig. 6f–h), which lasted through the post-HFS with no further depression. The HFS-mediated OFC-DMS pathway depression was also observed by comparing per-mouse averaged responses from noHFS (*n* = 7 mice) and HFS (*n* = 7 mice; Supplementary Fig. 5a) groups. Thus, protocols that induce plasticity in ex vivo preparations may not induce the same effects in vivo; and HFS can reliably depress OFC-DMS inputs in awake animals.

Recently, it has been shown that reduced excitability of orbito-striatal inputs between co-activated neuronal ensembles attenuates cocaine-induced psychomotor responses^24^. To understand whether opposite changes in orbito-striatal neuronal and input function bidirectionally modulate locomotion, we monitored animal’s velocity during and after Chronos-mediated optogenetic HFS of OFC, in absence of psychostimulants. During the first ON period (60 Hz stimulation for 5 min), we observed a dramatic increase in mouse speed (Supplementary Fig. 5b, c), confirming an association between heightened OFC-DMS pathway activity and hyperlocomotion. This result is also consistent with the OFC-DMS potentiation we observed with psychostimulants. In contrast, velocity decreased immediately after HFS, while it remained unaltered at the same time-point in the control group (Supplementary Fig. 5d). Thus, these data suggest that strengthening or weakening the OFC-DMS pathway might exert a bidirectional control over locomotion, even in the absence of cocaine.

### HFS of OFC-DMS blocks cocaine-induced potentiation and hyperlocomotion

**Fig. 7:**
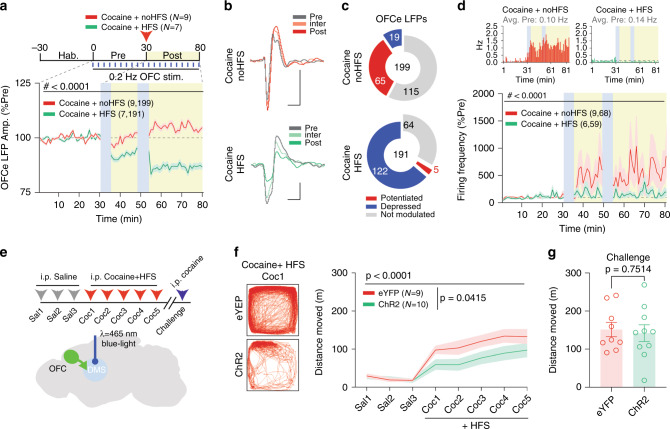
HFS overpowers cocaine-dependent OFC-DMS potentiation and attenuates hyperactivity. **a** Time-course of norm. OFCe LFP responses in mice exposed to cocaine before high-frequency stimulation (HFS) or no HFS (RM two-way ANOVA; time main effect: *F*_(69,26772)_  = 23.08, *p* < 0.0001; treatment main effect: *F*_(1,388)_ = 130.3, *p* < 0.0001; time Χ treatment interaction: *F*_(69,26772)_ = 71.29, *p* < 0.0001). **b** Representative OFCe LFP traces. Scale bar: 20 ms, 50 μV. **c** Pie-charts reporting the number of significantly potentiated or depressed and non-modulated OFCe LFP responses. **d** Time-course of norm. firing frequency of striatal multi-units in mice exposed to cocaine before HFS or no HFS (RM two-way ANOVA; time main effect: *F*_(73,9125)_ = 2.499, *p* < 0.0001; treatment main effect: *F*_(1,125)_ =  2.106, *p* = 0.1492; time Χ treatment interaction: *F*_(73,9125)_ = 1.756, *p* < 0.0001). Top: representative rate histograms. **e** Experimental schematics. **f** Mouse movement tracking examples during the first cocaine exposure (Coc 1) and distance moved during sensitization paradigm for eYFP and ChR2 infected mice (RM two-way ANOVA; time main effect: *F*_(7,119)_ = 28.91, *p* < 0.0001; virus main effect: *F*_(1,17)_ = 4.861, *p* = 0.0415; time Χ virus interaction: *F*_(7,119)_ = 2.009, *p* = 0.0595). **g** Distance moved upon cocaine challenge (unpaired two-tailed *t*-test: *t*_(17)_ = 0.3219). Data are represented as mean ±  SEM. *N*, *n* indicate number of mice and OFCe LFPs or units included in the analysis.

As cocaine potentiated the OFC-DMS connection and HFS depressed it, we asked whether the HFS protocol could block cocaine-induced increases in OFC-DMS connectivity, and associated increases in DMS activity. When we tested the effects of HFS applied to the OFC immediately after the cocaine injection (*n* = 7 mice), we observed that HFS overpowered the effect of cocaine and again depressed the OFCe LFP amplitude at post-HFS (Fig. 7a–c). Interestingly, in the no HFS group (*n* = 9 mice) we replicated our earlier experiments showing that cocaine increased the OFCe LFP amplitude (Fig. 7a–c). Again, this effect was also apparent at a per-mouse level when comparing no HFS vs HFS mice treated with cocaine (Supplementary Fig. 6a). HFS also blocked the cocaine-mediated increase in the average firing rate of striatal units (Fig. 7d), confirming a causal link between the cocaine-mediated potentiation of the OFC-DMS pathway and increases in neuronal activity in the striatum.

Finally, we examined the consequences of attenuating cortico-striatal drive on the sensitization of psychomotor responses to cocaine. For 5 consecutive days, mice bilaterally expressing either ChR2 or eYFP in the OFC and implanted with optic fibers in DMS (Fig. 7e) received i.p. injections of cocaine (20 mg/Kg) immediately followed by HFS. Consistent with our recording experiments, at the first cocaine exposure (Coc1), HFS of OFC inputs attenuated hyperactivity in ChR2- but not eYFP-expressing mice (Supplementary Fig. 6b). Although individual ChR2-expressing neurons likely cannot faithfully follow a 60 Hz stimulation train, we confirmed that ChR2-mediated stimulation increased power in the LFP at 60 Hz, confirming that the DMS population activity was modulated at that frequency (Supplementary Fig. 6c). Attenuation of locomotor responses was also observed over the next four consecutive cocaine injections (Fig. 7f). To test whether HFS at OFC-DMS was masking the expression of cocaine sensitization, these same animals received a cocaine challenge injection (without HFS) after 10 days of withdrawal from cocaine. Without HFS, ChR2- and eYFP-expressing mice displayed similar locomotor responses to this cocaine challenge (Fig. 7g). Thus, we conclude that HFS at OFC-DMS pathway acutely interfered with the expression of psychomotor responses to cocaine but did not prevent the acquisition of cocaine sensitization.

## Discussion

In the present study, exposure to cocaine increased the activity of both direct and indirect pathways in DMS. We linked these changes to a dopamine-dependent OFC input strengthening and a greater efficacy in driving the activity of DMS neurons. We tested several optogenetic stimulation protocols and found that high-frequency stimulation depressed OFC-DMS connectivity and reduced locomotion. HFS also over-powered the cocaine-induced OFC-DMS potentiation and, when applied at OFC-terminals, attenuated cocaine-induced hyperactivity in vivo.

Here, cocaine significantly increased the expression of c-fos in striatal regions, particularly in direct and indirect pathway DMS neurons, as reported by other studies^17,18^. Although our in vivo electrophysiology recordings showed a generalized increase in striatal neuron activity, they also revealed a smaller neuronal population that decreased its activity in response to cocaine, which might confirm important cellular specializations within striatal circuits^53,54^. However, this bidirectional change did not appear to be related to the two output pathways since photometry recordings revealed that cocaine increases the frequency of population calcium events in both. Thus, our results diverge from a classic model of striatal function by which psychostimulant-induced dopamine increase would respectively enhance and attenuate dMSN and iMSN activity via dopamine receptor activation. While this theory has been supported by evidence in ventral striatum^13^ and anesthetized animals^14^, cocaine was also reported to decrease population activity of both pathways in dorsal striatum^20^. Despite the discrepancy in the direction of change, our result is consistent with the Barbera et al.^20^ study in failing to provide evidence for a bidirectional action of cocaine on population activity in the direct and indirect pathways in awake mice. These inconsistencies point at anatomical, sub-regional or even cellular specializations in dopamine control of striatal pathway function which might be fundamental for a better understanding of the circuit adaptations underlying the neurobehavioral effects of psychostimulants.

In this study, we linked the increased activity of striatal pathways to a dopamine-dependent strengthening of orbito-striatal inputs, which resulted in a greater efficacy of OFC excitatory inputs at inducing post-synaptic neuron firing in awake animals. Given the abundance of intra-striatal collaterals^55,56^, we also found a small population of neurons that decreased their activity in response to OFC stimulation. The source of this inhibition is most likely a di-synaptic circuit composed by OFC inputs that excite intermediate GABA neurons that inhibit the recorded ones, which might not be directly activated by OFC. Although lateral inhibition plays an important role in behavioural responses to psychostimulants^16^, further investigation will clarify how cocaine modulates intra-striatal OFCe inhibitory responses.

The dopamine-dependent enhancement of OFC excitatory inputs that we observed is consistent with a previous observation that a reinforcing intra-cranial self-stimulation protocol causes a long-lasting increase in cortico-striatal post-synaptic potential in anaesthetized animals^57^. In addition, our data link the potentiation of OFC-inputs to downstream striatal neuron firing via possible dopamine-dependent changes in post-synaptic glutamatergic signal integration in real-time^21^. Considering that previous evidence showed a psychostimulant-mediated increase of glutamate in striatal regions, particularly upon sensitization^58^ and re-instatement of drug seeking behaviour^59^, the OFC-DMS potentiation might also be due to pre-synaptic adaptations. Possibly, these changes in neuronal integration and pre-synaptic glutamate release participate in the induction of the long-lasting adaptations observed at OFC-DMS synapses in response to repeated cocaine administration or dopamine neuron self-stimulation^24,28^.

While much effort focused on the PFC to NAc pathway stimulation to block drug-seeking behaviour in rodents^32,33^, the orbito-striatal pathway recently received attention for its role in compulsive seeking^28,60^ and locomotor sensitization to cocaine^24^. In our work, we mimicked classical stimulation protocols of cortico-striatal inputs to monitor and modulate OFC-DMS pathway function in awake animals, both during (ON) and after (OFF) LFS, TBS and HFS. During high-frequency stimulation, we observed an increase in animal’s velocity, indicating that strengthening OFC-DMS pathway acutely drives locomotion. This conclusion is corroborated by our observation of a prominent gamma-frequency oscillation in DMS, which is thought to be a prokinetic signal^61^, during cocaine-induced hyperactivity. Previous evidence that OFC-DMS inhibition attenuates cocaine-induced hyperlocomotion^24^ and that HFS increases axonal activity^62^, while promoting glutamate release both ex vivo^63,64^ and in vivo^65^, further supports the possibility that OFC-DMS potentiation leads to hyperactivity. However, whether OFC neurons fire action potentials in high-frequency patterns during locomotion bouts or upon cocaine exposure to enhance glutamate release warrants further investigation.

Upon termination of OFC-DMS stimulation (OFF period), HFS (but neither LFS nor TBS) modified orbito-striatal input strength. Therefore, our data indicate that HFS-induced LTD observed in both slice preparations^66^ and anesthetized animals^51^ also occurs in awake mice. Moreover, the failure of LFS or TBS to induce plasticity might indicate that optogenetic stimulation in vivo does not recruit the same molecular effectors as electrical stimulation ex vivo, as also suggested by previous observations^34^. The depression of OFC-DMS input following our HFS protocol might be due to multiple mechanisms, including the release of endocannabinoids that retrogradely inhibit glutamate release^66^, hetero-synaptic block of glutamate release by HFS-induced GABA release^67^ or long-term adaptations in the firing rates of stimulated neurons^68^.

High-frequency stimulation of OFC-DMS terminals decreased animal’s velocity both acutely and over consecutive days of cocaine exposure. In these settings, HFS restrained but did not prevent sensitization and did not block its expression at post-withdrawal cocaine challenge, which has been demonstrated to depend on NAc synaptic adaptations^69^. Our data directly link OFC-DMS potentiation to cocaine-induced hyperlocomotion, as well as increases in striatal neuron firing; however, this study does not address the effects of HFS on repeated saline injections, nor at the cocaine challenge. Therefore, it might be possible that HFS at OFC inputs onto DMS would decrease locomotor activity even in absence of cocaine exposure, as our data on HFS of OFC suggest. Further experimentation will be needed to evaluate this possibility. Finally, since excitatory synapse stimulation induces forms of hetero-synaptic plasticity^70^ and motor cortex stimulation can also promote LTD^51^ in striatal regions, the effects of HFS on other excitatory inputs to the striatum of awake mice and the resulting changes in behaviour also remain to be determined.

Monitoring the strength of the OFC-DMS connection in real time, as we did here, can link plasticity in specific brain circuits in animals to changes in connectivity observed in clinical studies^71,72^. Thus, this approach may be used in multiple circuits to prevent or rescue aberrant forms of plasticity, induced by drugs or diseases, via DBS or trans-cranial magnetic stimulation (TMS) protocols in both rodents and humans^32,34,35^.

## Methods

### Animals

Wildtype (WT), D1Cre (GENSAT: EY217), A2aCre (GENSAT: KG139)^73^ mice on a C57Bl6/j background, D1-tomato (JAX: B6.Cg-Tg(Drd1a-tdTomato)6Calak/J), and D2-GFP mice (Tg(Drd2-EGFP)S118Gsat) mice were used: 21 animals (12 males and 9 females) for in vivo electrophysiology experiments; 22 animals (12 males, 10 females) for fiber photometry, 20 animals (14 males and 6 females) for behavioral assessment of cocaine-induced hyperlocomotion, 6 animals (3 males and 3 females) for in vitro electrophysiology, and 8 males for phospho-c-Fos experiments. The animals were housed at the NIH research animal facility in standard vivarium cages with ad libitum food availability and 12-hour dark/light cycle. The experiments described here were conducted during light-period (typically between 9 a.m. and 7 p.m.). All experimental procedures were approved by the National Institute of Diabetes and Digestive and Kidney Diseases/National Institutes of Health Animal Care and Use Committee.

### Viral infusions and optic fiber implantation

Viral infections of OFC and DMS were conducted on adult male and female mice (older than 12 weeks). Anesthesia was induced with isoflurane at 2–3% and maintained during the entire surgery with isoflurane at 0.5–1.5%, delivered via a mouse mask mounted on a stereotaxic apparatus (Stoelting). Ear bars and mouth holder were used to keep the mouse head in place while the skin was shaved and disinfected with a povidone/iodine solution. The skull was exposed and a hole of ~0.5–1 mm diameter was performed with a microdrill. A 5 μL Hamilton syringe was connected to a 33gauge steel injector (Plastics1) via a hydraulic system. The injector was pre-loaded with AAVs and gently lowered into the brain at the following coordinates: OFC: AP + 2.5 mm, ML + 1.1 mm, DV −2.5 mm; DMS: AP + 0.5 mm, ML + 1.5 mm, DV −2.8 mm (from bregma). A total volume of 500 nl of viral solution was delivered at each infection site with a syringe pump (Harvard apparatus) at a rate of 50 nL min^−1^. The injector was left in place for 5 min after the infusion.

For in vivo electrophysiology experiments, the animals received a unilateral infection of the OFC. After removal of injector, suture (Coated VICRYL, polyglactin 910; Ethicon) followed by povidone/iodine were applied to close the wound. Animals were placed back in their home-cages on a pre-heated pad at 37 °C until complete recovery and subcutaneously injected with buprenorphine (0.05 mg Kg^−1^). Animals were allowed at least 2 weeks for viral expression before electrodes were implanted in a second surgery.

For fiber photometry experiments, immediately after unilateral viral infusion in DMS, a 5 mm long fiber optic cannulae (200 μm, 0.39 NA, 1.25 mm) with stainless steel ferrule (Thorlabs) was gently lowered into DMS between 0.5 and 0.3 mm above the infusion site for GCamP6s-emitted fluorescence recordings.

For behavioral experiments with cocaine and OFC-DMS input stimulation, mice received bilateral opsin viral infusions in the OFC. In the same surgery, two fiber optic cannulae (200 μm, 0.39 NA, 1.25 mm, ceramic ferrule) were lowered bilaterally into DMS for OFC terminal stimulation (AP + 0.5 mm, ML ± 1.5 mm, DV −2.8 mm).

Optic fibers were fixed to the skull with a layer of C&B Metabond® Quick Adhesive Cement System and the implant was fortified with acrylic dental cement. Upon solidification of the implant, animals were placed back in their home-cages on a pre-heated pad at 37 °C. After full recovery, animals received a subcutaneous injection of buprenorphine (0.05 mg Kg^−1^).

### Array and optic fiber implant for in vivo electrophysiology recordings

Implants of recording arrays for in vivo extracellular recordings and OFC stimulation typically occurred two weeks after viral infusions. In this study, we used 32-channel offset micro-arrays composed of 35 μm tungsten electrodes with polished tips and spaced by 150 μm. Uncleaved fiber optic cannulae (200  μm, 0.50 NA, 1.25 mm) with ceramic ferrule were purchased from Thorlabs and cut at the desired length for OFC stimulation. After anesthesia and skull exposure, two holes were drilled at the stereotaxic coordinates reported above. The optic fiber tip was lowered 0.3 mm above the infusion site with a 10° angle in the OFC, while the array was lowered into DMS. A grounding wire was inserted into the parietal lobe and a mini-screw was placed contro-laterally to the array to stabilize the implant. Both the optic fiber and the array were fixed to the skull and screw with C&B Metabond® Quick Adhesive Cement System and then cemented with dental acrylic. After surgery, animals were transferred to a pre-heated pad until complete recovery and subcutaneously injected with buprenorphine (0.05 mg  Kg^−1^). Mice were single-housed.

### Fiber photometry and calcium population activity analysis

About 2–4 weeks after surgery, mice (8 D1Cre, 6 A2aCre and 8 WT mice) of both sexes were acclimated for 30 min day^−1^ at least twice to a Phenotyper box (Noldus, PT T10/N, 24 VDC – 0.6 A) with dimensions 30 cm (l) ×30 cm (w) ×34 cm (h). On the experimental day, mice were recorded for 30 minutes, before receiving a saline or cocaine i.p. injection (20 mg Kg^−1^; counterbalanced). A mating sleeve (zirconia) connected the stainless-steel ferrule to the patch-cord (200 μm core optic fiber, 0.48 NA, Doric), which both transmitted excitatory blue-light (wavelength: 475 λm, power: ~30 μW) and collected GCaMP6s-emitted photons. An optical commutator (Doric) was used to allow for rotation of the mice without tangling. The emitted light passed through a dichroic mirror and 505–535 nm filter (FMC4 port minicube, Doric) and then measured with a photodetector (Model 2151, Newport). GCaMP6s signal was collected, digitized and measured with Omniplex acquisition system (Plexon, Inc). The change in fluorescence (dF) was normalized to total fluorescence (F). In fact, absolute fluorescence most likely reflects variance in viral expression and fiber micro positioning. Thus, before proceeding with further analysis and similarly to previous approaches^74^, we used a custom Python scripts run in NeuroExplorer (script available on request) to normalize the data and correct for bleaching in one step. Our custom-made script (available upon request) calculated a moving window of 2 min around each data point and used this as F, sliding this window along the entire recording trace to normalize each recorded data point and calculate a dF/F. Fluorescence events (>5% dF/F, with at least 1 s-long inter-event interval) were identified and their frequency binned in 60 s time intervals. A time-course was generated by normalizing 1-min bin frequency values to the average of 30 bins in the pre-injection period and expressed as percentage (% of Pre). Larger binned frequency values for pre- and post-injection periods were obtained by averaging the 1-min long bins within 11–30 min for Pre- and 41–60 min for post-injection periods. Animal movements in the open field were tracked and analyzed with Noldus Ethovision software.

### In vivo electrophysiology recordings

After recovery from surgery (between 7 and 10 days), mice were connected to the recording setup and acclimated to an open field, 34.5 cm (l) × 34.5 cm (w) × 34.5 cm (h), head-stage and cable for at least four sessions of 30 min each on different days. On the experimental day, multi and single-units were collected via an Omniplex neurophysiology system (Plexon Inc) through a multiplexing head-stage (Triangle Biosystems). Spike channels were acquired at 40 kHz with 16-bit resolution, and band-pass filtered at 150 Hz to 3 kHz before spike sorting, while LFP were digitized at 1 kHz. Single and multi-units were sorted by principal component analysis performed with Offline sorter software and MANCOVA test determined significant clustering of single-units vs multi-units. To be considered a single unit the spikes from that cluster must be significantly different (*p* < 0.01) from the multi-unit cluster on the same wire.

### In vitro electrophysiological recordings

Mice were C57BL/6 or heterozygous BAC transgenic mice in which tomato expression was driven by D1R (B6.Cg-Tg(Drd1a-tdTomato)6Calak/J from Jackson Laboratories) gene regulatory elements. AAV1-CAG-chR2-eYFP (UNC Viral Vector Core) was injected bilaterally into the OFC (350 μL, AP: +1.9 mm, ML: ±0.3 mm, DV: −2.5 mm) of 6-week-old mice. Following 28 days expression, coronal slices of the DMS (220 µM) were prepared using a vibratome in cutting solution containing: 76 mM NaCl, 26 mM NaHCO_3_, 75 mM sucrose, 25 mM glucose, 2.5 mM KCl, 1.25 mM NaH_2_PO_4_, 7 mM MgCl_2_ and 0.5 mM CaCl_2_. Slices were hemisected and transferred to ice cold artificial cerebrospinal fluid (ACSF) containing: 119 mM NaCl, 2.5 mM KCl, 1.3 mM MgCl_2_, 1.0 mM NaH_2_PO_4_, 26.2 mM NaHCO_3_, 2.5 mM CaCl_2_, glucose 11 mM, and continuously bubbled with 95 % O_2_ and 5 % CO_2_. Slices were submerged in 34 °C ACSF for 30 min and allowed to recover for another 30 min at room temperature. They were subsequently transferred to the recording chamber, superfused with 2.5 ml min^−1 ACSF at nearly physiological temperature (30–32 °C). Slices were visualized on a Nikon 600 N microscope equipped with a 40× objective lens. Neurons were recorded by means of whole-cell voltage-clamp at −68 mV, and excitatory post-synaptic currents were optically-evoked by flashing blue light (473 nm, 13.9–14.6 mW) through the light path of the objective, and isolated by recording in picrotoxin (100 µM, Sigma Biosciences). Borosilicate glass pipettes were prepared at a resistance range of 6–9 MΩ. The internal solution contained: CsCl 130 mM, 4 mM NaCl, 5 mM creatine phosphate, 2 mM MgCl_2_, 2.0 mM Na2ATP, 0.6 mM Na_3_GTP, 1.1 mM EGTA and 5 mM HEPES. Currents were amplified, filtered at 2 kHz and digitized at 10 kHz using Clampex 11 (pClamp, Molecular Devices). Access resistances were monitored by a hyperpolarizing step of −4 mV at the onset of every sweep and the experiment was discarded if the access resistance changed by more than 20%. ChR2 was stimulated by flashing 473 nm blue light (pulse length: 4 ms) through the light path of the microscope using an LED powered by an LED driver under computer control. Recordings were performed in aCSF, paired pulse ratios were evoked at an interval of 50 ms. Representative example traces are shown as average of 30 consecutive oEPSCs. Data were analyzed in Clampfit 11.3 software (pClamp, Molecular Devices).

### Pharmacological and optogenetic manipulations for in vivo electrophysiology recordings

Animals were exposed to pharmacological agents, optogenetic stimulation or both. Mice received intraperitoneal injections of psychostimulants (cocaine and amphetamine, and their respective controls), dopaminergic antagonists (SCH23390, Sulpiride and their respective vehicle controls) and GBR13069 (and its control). In vivo plasticity was tested via the delivery of blue-light pulses into the OFC at different frequencies: low-frequency stimulation (LFS) consisted in 15 min-long delivery of 15 ms-long blue-light pulses at 5 Hz; theta-burst stimulation (TBS) consisted in the repeated delivery (10 times) of 10 trains of action potentials (at theta-frequency: 10.5 Hz) organized in 4 light-pulses at 50 Hz and HFS consisted in the delivery of 2-blocks of 5-min long 60 Hz stimulation interleaved by a 14 min interval.

### Analysis of in vivo electrophysiology recordings

The firing frequency of multi- and single-units during pre- and post-manipulation period was calculated in 60-s bins with Neuroexplorer software. Average firing frequencies for pre- and post-manipulation periods were calculated as average of 30 bins in pre- and 30 bins in post-manipulation periods. A paired *t*-test followed by Bonferroni correction (*α* = 0.05 n^−1^ of comparisons) was performed between pre- and post-manipulation period (10–30 min bin) to determine whether the manipulation induced significant changes in firing frequencies. To determine the direction of the change the average firing frequency during the post-manipulation was expressed as percentage of the pre-manipulation, such that changes >100% identified an increase while changes <100% indicated a decrease in firing frequency.

For OFC-stimulation evoked (OFCe) firing frequency, animals were subjected to 15 ms long blue-light pulses at 0.2 Hz, 30 min before and 30 min after pharmacological or optogenetic stimulation. The power of the laser was adjusted between 8 and 30 mW to obtain reliable responses. To determine significant changes in neuronal activity, firing frequency was analyzed 25 ms before (OFF period) and 25 ms after the beginning of OFC illumination (ON period). A paired t-test between trial-by-trial values of firing frequency was used to determine significant modulation by OFC stimulation. A paired *t*-test followed by Bonferroni correction between trial-by-trial (25 ms after the beginning of blue-light stimulation) during pre- (1–30 mins) and post-manipulation (31–60 mins) periods was used to determine significant modulation in OFCe firing rates. A time-course of OFCe firing frequency was generated by binning OFCe firing (60 s) and expressing it as percentage of the averaged OFCe firing during pre-manipulation period. Binned OFCe firing rate responses were obtained by averaging 60-sec bins for pre- and post-manipulation period.

For OFC-stimulation evoked local-field potentials (OFCe LFPs) responses, animals were subjected to 15 ms long blue-light delivery into OFC at 0.2 Hz, 30 minutes before (Pre) and 30 min after (Post) pharmacological or optogenetic manipulations. The power of the laser was adjusted between 8 and 30 mW to obtain reliable responses. OFCe LFP amplitude was measured by using a custom-made Python script as the difference between the minimum and a maximum deflection point within an interval of 100 ms before and after OFC stimulation. To determine significantly OFCe LFP responses, a trial-by-trial paired *t*-test on the peak-to-peak amplitude in each trial before (100 ms) and after (100 ms) the blue-light pulse in the pre- phase was conducted. The amplitude of significant OFCe LFP responses was binned in 60-s bins and expressed as percentage of averaged OFCe LFP amplitude during pre-manipulation period. Significantly modulated OFCe LFP responses were identified by paired t-test followed by Bonferroni correction (*α* = 0.05 n^−1^ of comparisons). To determine the direction of the change of OFCe LFP responses, the averaged OFCe LFP amplitude during the post-manipulation period was expressed as percentage of the averaged OFCe LFP amplitude in pre-manipulation period, such that changes >100% identified an increase while changes  <100% indicated a decrease OFCe LFP amplitude. Binned OFCe LFP responses were obtained by averaging 60-s bins for pre- and post-manipulation period. Per-mouse analysis of OFCe LFPs was conducted by averaging the change of OFCe LFPs within each animal.

Power spectra analysis was performed by applying Bartlett single-taper, on 512 frequency values from 0 to 100 Hz and a window overlap of 50. For each electrode, the power was expressed as a percentage of total and then averaged by mouse. Per-mouse power spectral densities were obtained by averaging power spectral densities for saline and cocaine injected mice. Binned power analysis was performed by binning and averaging power spectra values per-mouse as follows: delta (0–4 Hz), theta (4–10 Hz), beta (10–30 Hz), low-gamma (30–80 Hz) and high-gamma (80–100 Hz).

The averaged number of multi-units, OFCe LFPs and OFC-modulated units per mouse (together with standard deviation) included in each data-set is reported in Supplementary Table 1.

Animal movement in the arena was tracked via Omniplex system software Cineplex. X and Y coordinates were used to determine instant velocity (1 s). Instant velocity was averaged and binned (60 s) and extracted via Neuroexplorer. Binned velocity in post-manipulation period was then normalized to the averaged binned velocity in pre-manipulation period, to generate time-course of normalized velocity (expressed as a percentage of Pre).

### Behavioral assay of sensitization to cocaine

The sensitization of psychomotor responses to cocaine was tested in an open-field box of 30 cm (l) × 30 cm (w) × 43 cm (h). eYFP or ChR2-expressing animals were first habituated to the novel environment and patch-cord for 3 consecutive days. On habituation days, animals were acclimated to the box for 30 min before receiving an i.p. injection of saline (without any optical stimulation). Starting from day 4, animals were acclimated to the box for 30 min before receiving an i.p. injection of cocaine (20 mg Kg^−1^) immediately followed by HFS, with a laser power of 8 mW measured at the tip of the optic fiber. This protocol was repeated for 4 more consecutive days, for a total of 5 days of cocaine + HFS. After 10 days of withdrawal, animals were acclimated to the box for 30 min and received an i.p. injection of cocaine without any optic stimulation. Animal’s activity (distance moved and locomotion) was tracked with Ethovision software. Distance moved was calculated as the sum of binned distance moved during the 14 minutes inter-HFS and 16 min post HFS. We excluded from the analysis a ChR2-expressing animal that at day 1 showed a distance moved higher than 3 standard deviations from the group mean.

### Drugs

Cocaine hydrochloride (20 mg Kg^−1^; NDC 51552-0881-1, Fagron) and d-amphetamine hemisulfate salt C-IIN (3 mg Kg^−1^; A5880, Sigma) were obtained via NIH pharmacy and dissolved in saline (NaCl 0.9%) to perform intra-peritoneal (i.p.) injection of 200–300 μL. (S)-(-)-Sulpiride (25 mg Kg^−1^; 0895, Tocris), SCH23390 (lowSCH: 0.03 mg Kg^−1^; highSCH: 0.15 mg Kg^−1^; 0925, Tocris) and GBR13069 dihydrochloride (20 mg Kg^−1^; 0420, Tocris) were diluted in a vehicle solution of 10% DMSO in saline for i.p. injection.

### Viruses

rAAV2/Syn-Chronos-GFP (2.1 × 10^12^ vg mL^−1^), rAAVDJ/PAAV-Ef1a-DIO-GCaMP6s (3.9 × 10^12^ vg mL^−1^) and rAAV2/hSyn-eYFP (3.4 × 10^12^ vg mL^−1^) were purchased from Virus Vector Core at University of North Carolina at Chapel Hill. AAV5.CAG.hChR2(H134R)-mCherry.WPRE.SV40 (Addgene20938M, 3.9 ×  10^12^ GC mL^−1^) was purchased from Addgene.

### Phospho-fos experiments, immunostaining, and cell counting

Mice (5 D1-tmt and 3 D2-gfp) were sacrificed 2 hours after an i.p. injection of cocaine (20 mg Kg^−1^) or saline (*n* = 4 mice each). Brains were extracted and sectioned at 40 μm on a vibratome (Precisionary Compressotome). Tissue slices containing the striatum were immunostained for phospho-c-Fos (Cell Signaling monoclonal antibody #5348), with fluorescent secondary antibodies (Alexa 488 for D1-tmt mice and Alexa 555 for D2-gfp mice).

For quantification of striatal expression area, two striatal hemispheres per mouse were imaged on a scanning epifluorescence microscope (Leica DM6B). Phospho-c-Fos positive nuclei were identified manually in ImageJ and their X and Y coordinates were exported, registered to a common Atlas space, and plotted as heatmaps with MatPlotLib in Python 3.7. For colocalization with D1-tmt or D2-gfp, six two-color 10× fields of view were acquired from the DMS for each mouse on a confocal microscope (Leica microsystems). Images were quantified in ImageJ using the CellCounter plugin, where each phospho-c-Fos positive nucleus was also scored as positive or negative for the complementary fluorophore for cell type identification. To quantify the relative co-localization with D1R and D2R-expressing MSNs, confocal images were only acquired if they included multiple Fos positive nuclei (mean = 16.2, range = 3–32 Fos positive nuclei). This approach over-sampled Fos positive nuclei in the images from the saline group. We therefore normalized the number of co-labeled neurons by the total Fos in the striatum of each mouse to obtain the extrapolated total counts in Fig. 2e.

### Histological verification of sites of implant

At the end of the experiments, we performed a histological verification of implant placement. Animals were anesthetized (Chloral Hydrate, 7%) and perfused with 4% formaline. After over-night incubation in a 30% sucrose solution, either coronal or sagittal brain slices containing OFC and DMS were prepared. Slices were mounted on microscope slides with a mounting media with Fluoromount-G^TM^, with DAPI (Invitrogen) and imaged with a confocal microscope (Zeiss). For in vivo electrophysiology, behavioral and fiber photometry experiments implant placement was assessed via observation of implant tract or electric-lesions (performed with a 5-s long pulse of 10 mA; Ugo Basile Lesion Making Device).

### Statistical analysis

The number of animals included in this study was chosen based on those used in similar publications. Shapiro-Wilk test was used to assess normality of two-sample distributions. If violated, Mann–Whitney and Wilcoxon matched-pairs signed rank tests were applied, otherwise two-sided unpaired or paired *t*-tests were used. For independent multiple-sample distribution comparisons, one-way ANOVA or non-parametric Kruskal–Wallis tests were applied and followed by between-subject Bonferroni (parametric), one-sided *t*-test (parametric) or Dunn’s (non-parametric) post hoc test. For comparing two-factors multiple-sample distributions, normality of sample distribution was assumed and repeated-measures (RM) ANOVA, two-way ANOVA or RM two-way ANOVA were applied. When sphericity was not assumed, Geisser-Greenhouse correction was applied. If main effects and/or interaction were significant, between- or within-subject Bonferroni post-hoc or Dunnett’s tests were used to determine significant differences across sample distributions. For the analysis of the by-mouse expression of c-Fos we applied the post-hoc False Discovery Rate method of Benjamini, Krieger and Yekuteli test. In all tests, statistical significance was determined when *p* < 0.05 and n.s. indicates a non-significant difference between sample distributions (*p* > 0.05). Data are expressed as mean ± SEM. GraphPad Prism 8 software was used for graph presentation and statistical analysis. Statistical analysis is reported in figure legends. On graphs, p refers to *p*-values of main effects, comparison between two-sample distributions or post hoc tests, while # refers to p-values of two factor interaction upon RM or regular two-way ANOVA. Animals were randomly assigned to each experimental condition, but experimenters were not blinded to the treatment conditions as stimulants produced obvious behavioral changes upon injection.

### Reporting summary

Further information on research design is available in the Nature Research Reporting Summary linked to this article.

## Supplementary information

Supplementary Information

Peer Review File

Supplementary Data 1

Reporting Summary

## Data Availability

The data included in this paper are provided as Source data files and available at https://osf.io/dup4r/.

## Code availability

Python codes used for the analysis of the data included in this manuscript are provided as Supplementary Software and available at: https://osf.io/dup4r/.
