# Supplementary material for: Orbitofrontal-striatal potentiation underlies cocaine-induced hyperactivity
Source: Nat Commun. 2020 Aug 10;11:3996. doi: 10.1038/s41467-020-17763-8 (PMC7417999; doi:10.1038/s41467-020-17763-8)
Supplement: Supplementary file 1 — Supplementary Information [file 41467_2020_17763_MOESM1_ESM.pdf]

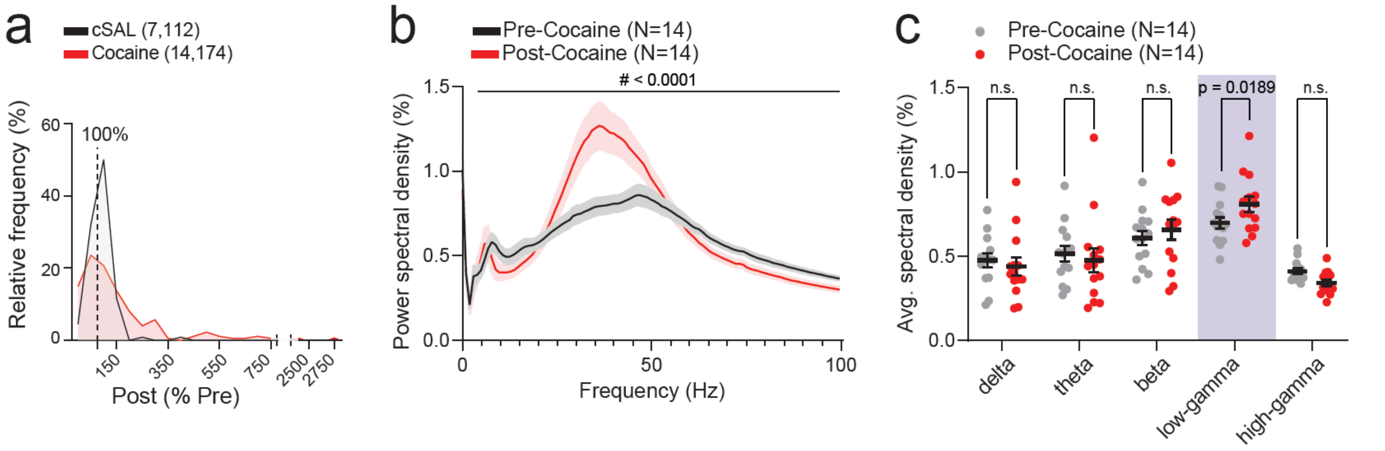

Supplementary Figure 1

Supplementary Figure 1. Firing rates and field-potential oscillations in DMS

(a) Relative frequency distribution of multi-unit activity change (% of Pre) upon saline (cSAL) or cocaine injection. (b) Averaged power spectra for cocaine and saline treated animals (RM two-way ANOVA; rhythms main effect: $F_{(102,1326)} = 28.09$, $p < 0.0001$; drug main effect: $F_{(1,13)} = 29.64$, $p = 0.0001$; rhythms X drug interaction: $F_{(102,1326)} = 12.49$, $p < 0.0001$). (c) Binned analysis of power spectra during pre- and post-cocaine periods (RM two-way ANOVA; rhythms main effect: $F_{(4,52)} = 14.54$, $p < 0.0001$; drug main effect: $F_{(1,13)} = 0.0189$, $p = 0.8927$; rhythms X drug interaction: $F_{(4,52)} = 4.287$, $p = 0.0045$; followed by between-subject Bonferroni post-hoc test cSAL vs COC; delta $t_{(52)} = 1.014$; theta $t_{(52)} = 1.091$; beta $t_{(52)} = 1.378$; low-gamma $t_{(52)} = 3.033$; high-gamma $t_{(52)} = 1.963$). Data are represented as mean ± SEM and/or single values. N,n indicate number of mice and units included in the analysis.

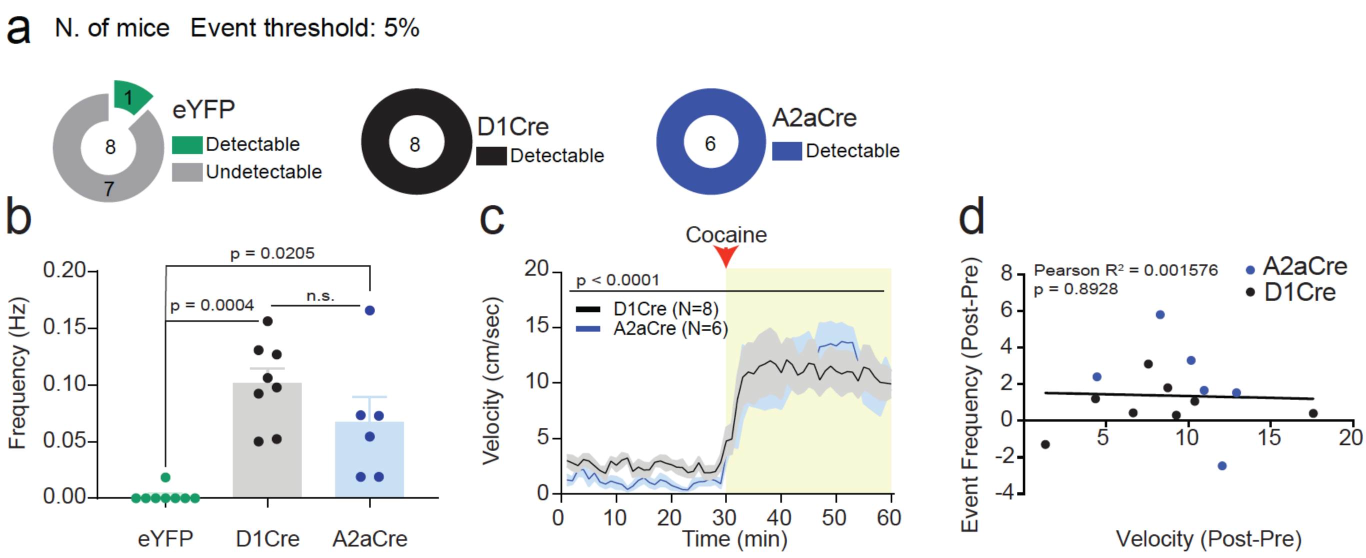

Supplementary Figure 2

Supplementary Figure 2. Frequency of calcium events in D1Cre and A2aCre mice
(a) Pie-chart reporting the number of WT-EYFP, D1-Cre and A2a-Cre animals with photometry events with local-maxima higher that 5% dF/F. (b) Frequency of dF/F events (> 5%) in WT, D1-Cre and A2a-Cre animals (Kruskal-Wallis, K = 15.81; followed by between-subject Dunn's test: EYFP vs D1-Cre Z = 3.835, EYFP vs A2a-Cre Z = 2.705; D1-Cre vs A2aCre Z = 0.8454). (c) Time-course of velocity for D1-Cre and A2a-Cre mice injected with cocaine (RM two-way ANOVA; time main effect: $F_{(2.809,33.71)} = 31.11$, $p < 0.0001$; genotype main effect: $F_{(1,12)} = 0.2283$, $p = 0.6414$; time X genotype interaction: $F_{(59,708)} = 0.8194$, $p = 0.8304$). (d) Correlation analysis of calcium event frequency and velocity changes upon cocaine injection in D1-Cre and A2a-Cre mice (Pearson $R^2 = 0.001576$, $p = 0.8928$). Data are represented as mean ± SEM and/or single values. N,n indicate number of mice and units included in the analysis.

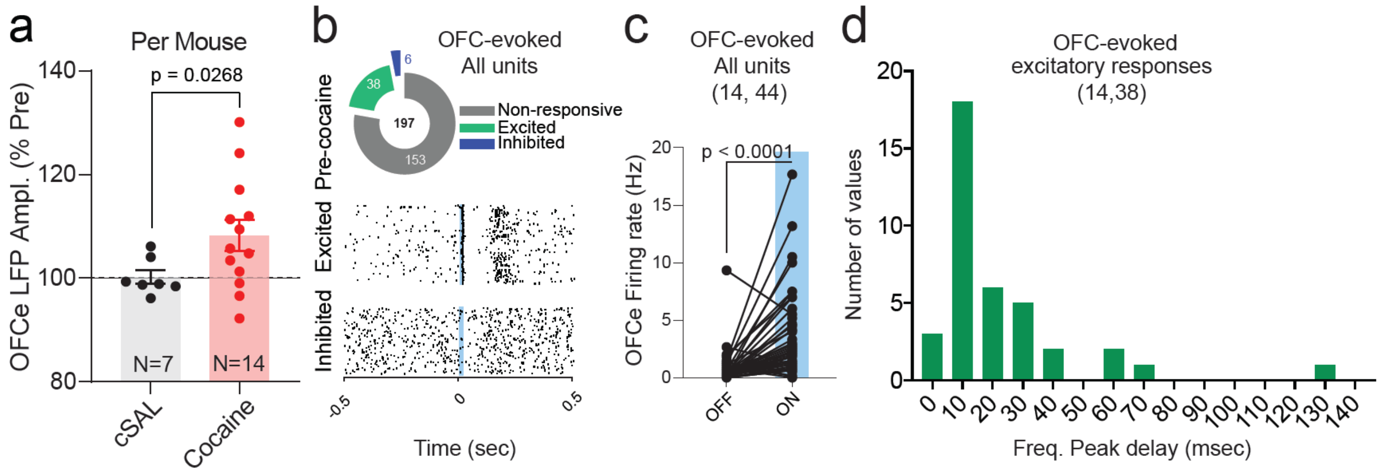

Supplementary Figure 3

Supplementary Figure 3. OFC stimulation primarily evokes excitatory striatal responses
(a) Per-mouse averaged norm. OFCe LFP responses in saline (cSAL) and cocaine injected mice (unpaired t-test, $t_{(15.96)} = 2.439$). (b) Pie-charts with example raster-plots of striatal units excited or inhibited by OFC stimulation. (c) OFCe firing rate before (OFF) and after OFC stimulation (ON; Wilcoxon matched-pairs signed rank test: W = 823.0). (d) Frequency distribution of peak latency of OFC evoked responses. Data are represented as single points and/or as mean ± SEM. N,n indicate number of mice and units included in the analysis.

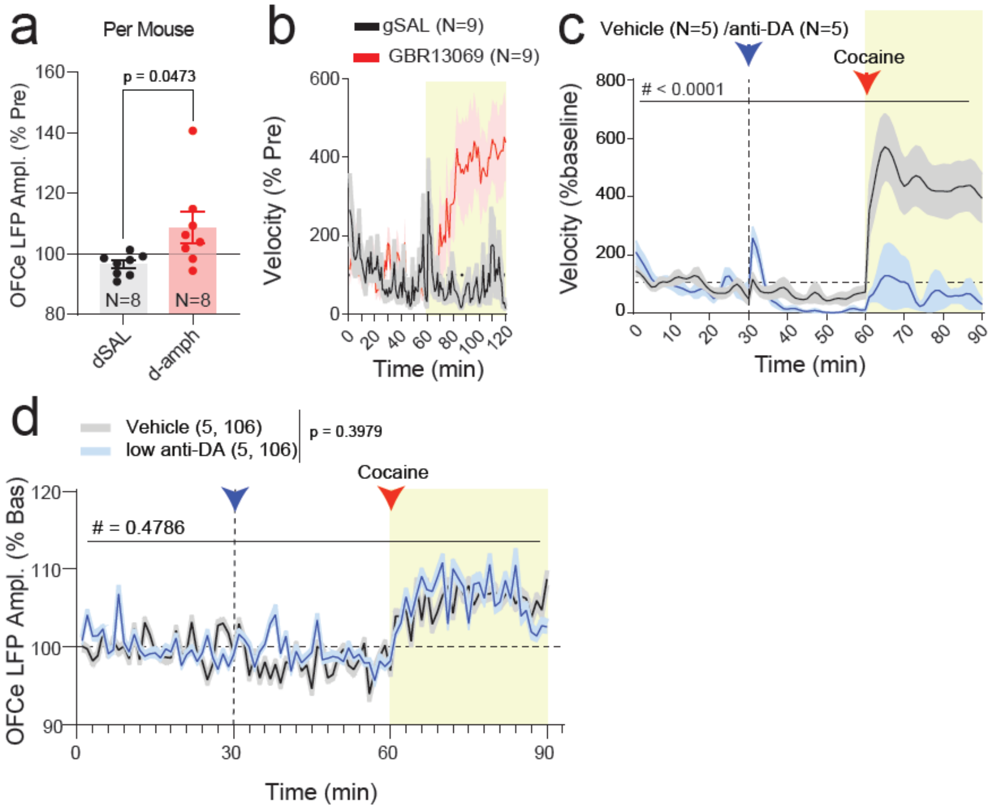

Supplementary Figure 4

Supplementary Figure 4. Locomotor effects of GBR13690 and anti-DA pre-treatment

(a) Per-mouse norm. OFCe LFP responses in saline (dSAL) and d-amphetamine (d-amph) injected mice (unpaired t-test, $t_{(7.872)}$ = 2.348). (b) Time-course of norm. velocity upon saline (gSAL) or GBR13069 i.p. injection (RM two-way ANOVA; time main effect: $F_{(4.840,77.44)}$ = 3.588, p = 0.0062; drug main effect: $F_{(1,16)}$ = 9.044, p = 0.0084; time X drug interaction: $F_{(119,1904)}$ = 4.656, p < 0.0001). (c) Time-course of norm. velocity in mice exposed to cocaine and pre-treated with either vehicle or anti-DA (RM two-way ANOVA by both factors; time main effect: $F_{(89,356)}$ = 11.27, p < 0.0001; treatment main effect: $F_{(1,4)}$ = 14.47, p = 0.0190; time X treatment interaction: $F_{(89,356)}$ = 8.964, p < 0.0001). (d) Time-course of norm. OFCe LFPs in mice exposed to cocaine and pre-treated with either vehicle or low anti-DA (RM two-way ANOVA; time main effect: $F_{(2,210)}$ = 94.80, p < 0.0001; treatment main effect: $F_{(1,105)}$ = 0.7204, p = 0.3979; time X treatment interaction: $F_{(2,210)}$ = 0.7396, p = 0.4786). Data are represented as single points and/or as mean ± SEM. N, n indicates number of mice and OFCe LFPs included in the analysis.

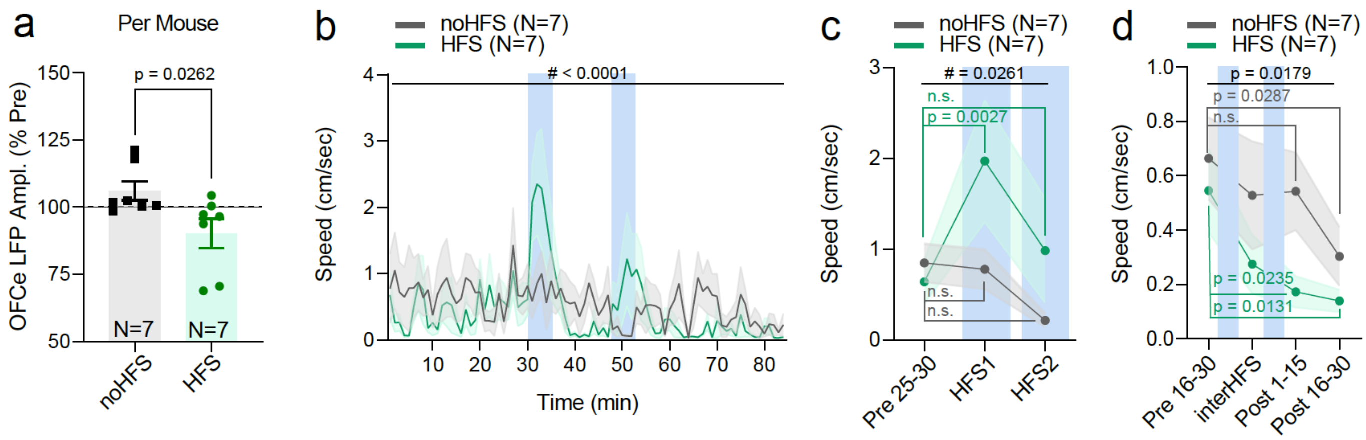

Supplementary Figure 5

Supplementary Figure 5. Locomotor effects of HFS stimulation
(a) Per-mouse norm. OFCe LFP amplitude in high-frequency stimulation (HFS) or noHFS treated mice (Mann Whitney test, U = 7). (b) Time-course of animal's speed before, during and after noHFS and HFS (RM two-way ANOVA; time main effect: $F_{(83,498)} = 2.177$, $p < 0.0001$; protocol main effect: $F_{(1,6)} = 0.6411$; time X protocol interaction: $F_{(83,498)} = 1.807$, $p < 0.0001$). (c) Binned animal's speed before, and during the first and second HFS periods in noHFS and HFS (RM two-way ANOVA; time main effect: $F_{(2, 12)} = 6.412$, $p = 0.0128$; protocol main effect: $F_{(1, 6)} = 1.687$, $p = 0.2417$; time X protocol interaction: $F_{(2, 12)} = 5.016$, $p = 0.0261$; followed by within-subject Bonferroni post-hoc test for noHFS group: pre25-30 vs HFS1 $t_{(12)} = 0.2198$ and $p > 0.9999$, pre25-30 vs HFS2 $t_{(12)} = 1.98$ and $p = 0.1423$; and HFS group: pre25-30 vs HFS1: $t_{(12)} = 4.146$ and $p = 0.0027$, pre25-30 vs HFS2: $t_{(12)} = 1.07$ and $p = 0.6114$). (d) Binned animal's speed before, between and after HFS stimulation in noHFS and HFS (RM two-way ANOVA; time main effect $F_{(3,18)} = 4.359$, $p = 0.0179$; protocol main effect $F_{(1,6)} = 2.244$, $p = 0.1848$, time X protocol interaction: $F_{(3,18)} = 0.7920$, $p = 0.5141$; followed by within-subject Bonferroni post-hoc test for noHFS group: pre16-30 vs interHFS $t_{(18)} = 1.094$, pre16-30 vs post1-15 $t_{(18)} = 0.9754$, pre16-30 vs post16-30 $t_{(18)} = 2.898$; and HFS group: pre16-30 vs interHFS $t_{(18)} = 2.172$, pre16-30 vs post1-15 $t_{(18)} = 2.992$, pre16-30 vs post 16-30 $t_{(18)} = 3.258$). Data are represented as single points and/or as mean ± SEM. N indicates number of mice included in the analysis. Data are represented as single points and/or as mean ± SEM. N indicates number of mice included in the analysis.

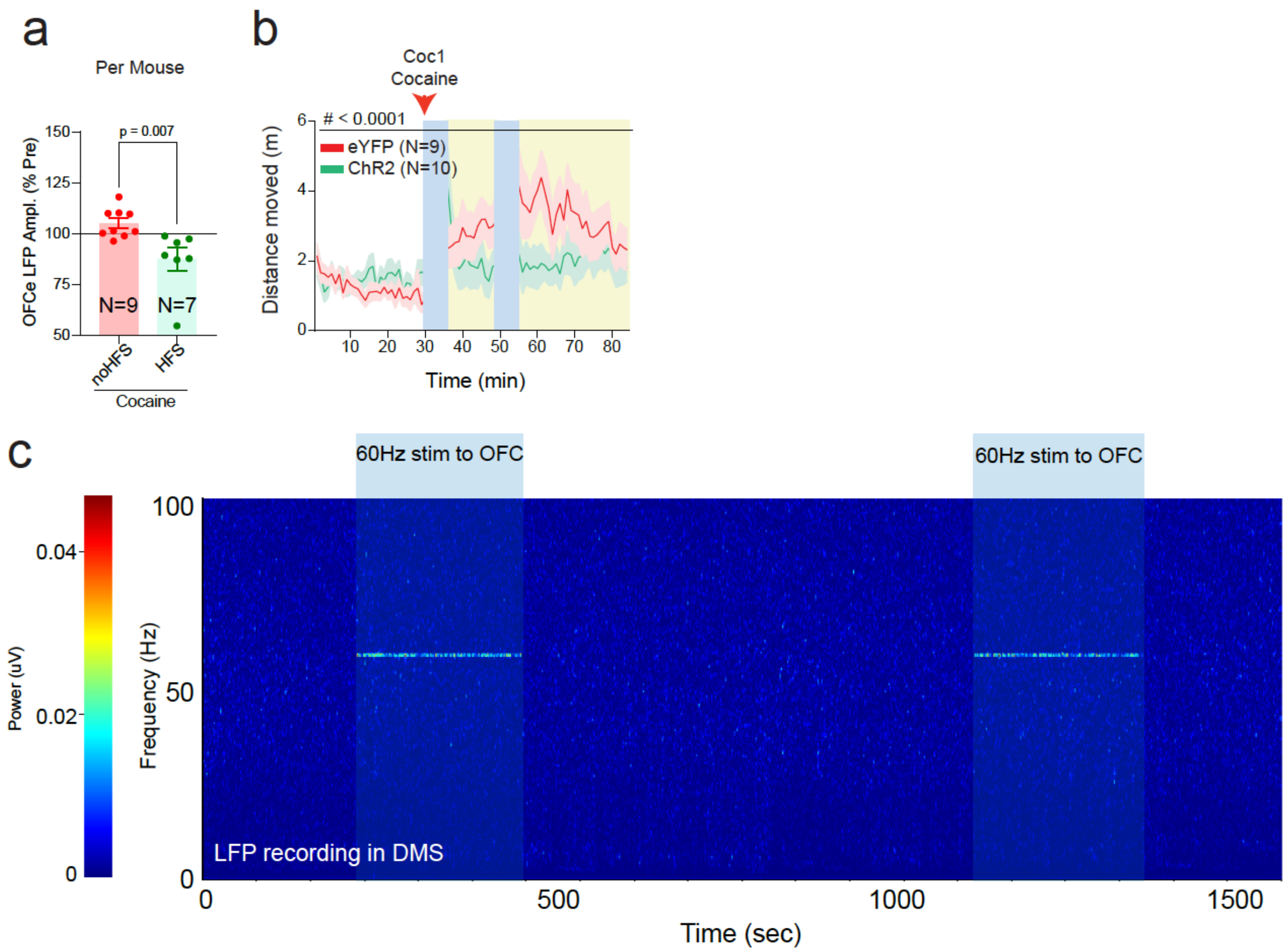

Supplementary Figure 6

Supplementary Figure 6. Locomotor effects of HFS on cocaine exposure

(a) Per-mouse norm. OFCe LFP amplitude in high-frequency stimulation (HFS) or noHFS mice treated with cocaine (Mann Whitney, U = 2). (b) Time-course of distance moved at first cocaine exposure (Coc1) during cocaine sensitization paradigm for eYFP and ChR2 HFS mice. (RM two-way ANOVA; time main effect: $F_{(73,1241)} = 3.596$, $p < 0.0001$; virus main effect: $F_{(1,17)} = 2.210$, $p = 0.1555$; time X virus interaction: $F_{(73,1241)} = 1.728$, $p = 0.0002$). (c) Spectrogram showing increase in LFP power at 60Hz in the DMS during HFS stimulation of the OFC. Data are represented as single points and/or as mean ± SEM. N indicates number of mice included in the analysis.

	Experiment	Average	Standard deviation
Figure 1	Saline multi-unit	16.0	7.2
Figure 1	Cocaine multi-unit	12.4	5.4
Figure 3	Saline OFCe LFP	21.7	9.0
Figure 3	Cocaine OFCe LFP	22.3	9.6
Figure 3	Coaine OFCe Firing	5.4	5.6
Figure 4	dSAL OFCe LFP	18.8	9.3
Figure 4	d-AMPH OFCe LFP	20.6	10.2
Figure 4	gSAL OFCe LFP	25.9	8.3
Figure 4	GBR OFCe LFP	26.4	7.7
Figure 5	Veh - Cocaine OFCe LFP	20.6	6.7
Figure 5	antiDA - Cocaine OFCe LFP	21.2	10.7
Figure 6	noLFS OFCe LFPs	21.0	9.5
Figure 6	LFS OFCe LFPs	17.1	10.5
Figure 6	noTBS OFCe LFPs	20.0	11.6
Figure 6	TBS OFCe LFPs	23.3	9.5
Figure 6	noHFS OFCe LFPs	27.9	4.1
Figure 6	HFS OFCe LFPs	29.0	4.5
Figure 7	COC - noHFS OFCe LFPs	22.1	9.8
Figure 7	COC - HFS OFCe LFPs	27.3	4.7
Figure 7	COC - noHFS multi-unit	7.8	5.4
Figure 7	COC - HFS multi-unit	9.8	7.0

Supplementary Table 1. Number of multi-units, OFCe LFPs and OFCe units per mouse
Averaged number and standard deviation of multi-units, OFCe LFPs and OFCe units recorded from each experimental subject (mouse) in each experimental dataset (experiment).
