# Supplementary material for: Orbitofrontal-striatal potentiation underlies cocaine-induced hyperactivity
Source: Nat Commun. 2020 Aug 10;11:3996. doi: 10.1038/s41467-020-17763-8 (PMC7417999; doi:10.1038/s41467-020-17763-8)
Supplement: Supplementary file 4 — Reporting Summary [file 41467_2020_17763_MOESM4_ESM.pdf]

nature research

Corresponding author(s): Alexxai Kravitz

Last updated by author(s): May 7, 2020

Reporting Summary

Nature Research wishes to improve the reproducibility of the work that we publish. This form provides structure for consistency and transparency in reporting. For further information on Nature Research policies, see our Editorial Policies and the Editorial Policy Checklist.

Statistics

For all statistical analyses, confirm that the following items are present in the figure legend, table legend, main text, or Methods section.

n/a	Confirmed	
☐	☒	The exact sample size (*n*) for each experimental group/condition, given as a discrete number and unit of measurement
☐	☒	A statement on whether measurements were taken from distinct samples or whether the same sample was measured repeatedly
☐	☒	The statistical test(s) used AND whether they are one- or two-sided<br>*Only common tests should be described solely by name; describe more complex techniques in the Methods section.*
☐	☒	A description of all covariates tested
☐	☒	A description of any assumptions or corrections, such as tests of normality and adjustment for multiple comparisons
☐	☒	A full description of the statistical parameters including central tendency (e.g. means) or other basic estimates (e.g. regression coefficient) AND variation (e.g. standard deviation) or associated estimates of uncertainty (e.g. confidence intervals)
☐	☒	For null hypothesis testing, the test statistic (e.g. *F*, *t*, *r*) with confidence intervals, effect sizes, degrees of freedom and *P* value noted<br>*Give P values as exact values whenever suitable.*
☒	☐	For Bayesian analysis, information on the choice of priors and Markov chain Monte Carlo settings
☒	☐	For hierarchical and complex designs, identification of the appropriate level for tests and full reporting of outcomes
☒	☐	Estimates of effect sizes (e.g. Cohen's *d*, Pearson's *r*), indicating how they were calculated

Our web collection on statistics for biologists contains articles on many of the points above.

Software and code

Policy information about availability of computer code

Data collection	GCaMP6s signal was collected, digitized and measured with Omniplex acquisition system (Plexon, Inc). Local field potentials, multi and single-units were collected via an Omniplex neurophysiology system (Plexon Inc) through a multiplexing head-stage (Triangle Biosystems). Ethovision (Noldus) software was utilized for behavioral experiments.
Data analysis	For fiber photometry experiments, the change in fluorescence (dF) was normalized to total fluorescence (F) using a custom Python scripts run in NeuroExplorer (script available on request). This script calculated a moving window of 2 minutes around each data point and used this as F, sliding this window along the entire recording trace to normalize each recorded data point. This approach normalizes the data and corrects for bleaching in one step.<br>Electrophysiology recordings were analyzed via Neuroexplorer Software after spike sorting via Offline Sorter software. A Python script identifying a maximum and a minimum value within 50 msec after blue-light pulse was generated for a trial-by-trial analysis of LFP amplitude and statistic. A python script was used to generate time-course for OFC-evoked firing and to determine statistical significance.

For manuscripts utilizing custom algorithms or software that are central to the research but not yet described in published literature, software must be made available to editors and reviewers. We strongly encourage code deposition in a community repository (e.g. GitHub). See the Nature Research guidelines for submitting code & software for further information.

Data

Policy information about availability of data

All manuscripts must include a data availability statement. This statement should provide the following information, where applicable:

- Accession codes, unique identifiers, or web links for publicly available datasets
- A list of figures that have associated raw data
- A description of any restrictions on data availability

The data that support the findings of this study are available from the corresponding author upon reasonable request.

Field-specific reporting

Please select the one below that is the best fit for your research. If you are not sure, read the appropriate sections before making your selection.

☒ Life sciences ☐ Behavioural & social sciences ☐ Ecological, evolutionary & environmental sciences

For a reference copy of the document with all sections, see nature.com/documents/nr-reporting-summary-flat.pdf

Life sciences study design

All studies must disclose on these points even when the disclosure is negative.

Sample size	The number of animals included in this study was chosen based on those used in similar publications.
Data exclusions	We excluded one animal from behavioral analysis because it was deviating more than 3 standard deviation from the group in activity in the baseline condition (detailed in Material and Methods).
Replication	Cocaine potentiation of OFC-DMS inputs was replicated at least twice in the manuscript. The experiments were conducted in batch of 3-5 animals, so each of them can be considered as an independent sample group and replication.
Randomization	Animals were randomly assigned to each experimental group.
Blinding	Experimenters were not blind to the treatment because of the obvious stimulant effect of dopaminergic drugs.

Reporting for specific materials, systems and methods

We require information from authors about some types of materials, experimental systems and methods used in many studies. Here, indicate whether each material, system or method listed is relevant to your study. If you are not sure if a list item applies to your research, read the appropriate section before selecting a response.

Materials & experimental systems

n/a	Involved in the study	
☐	☐	Antibodies
☒	☐	Eukaryotic cell lines
☒	☐	Palaeontology and archaeology
☐	☒	Animals and other organisms
☒	☐	Human research participants
☒	☐	Clinical data
☒	☐	Dual use research of concern

Methods

n/a	Involved in the study	
☒	☐	ChIP-seq
☒	☐	Flow cytometry
☒	☐	MRI-based neuroimaging

Antibodies

Antibodies used	phospho-c-Fos (Cell Signaling monoclonal antibody #5348), with fluorescent secondary antibodies (Alexa 488 for D1-tmt mice and Alexa 555 for D2-gfp mice).
Validation	The antibody used are commercially available and the validation has been performed by the seller.

Animals and other organisms

Policy information about studies involving animals; ARRIVE guidelines recommended for reporting animal research

Laboratory animals	Wildtype (WT), D1Cre (GENSAT: EY217), A2aCre (GENSAT: KG139)76 mice on a C57Bl6/j background, D1-tomato (JAX: B6.Cg-Tg(Drd1a-tdTomato)6Calak/J), and D2-GFP mice (Tg(Drd2-EGFP)S118Gsat) mice were used: 21 animals (12 males and 9 females) for

in vivo electrophysiology experiments; 14 animals (9 males, 5 females) for fiber photometry, 20 animals (14 males and 6 females) for behavioral assessment of cocaine-induced hyperlocomotion, 6 animals (3 males and 3 females) for in vitro electrophysiology, and 8 males for phospho-c-Fos experiments. The animals were housed at the NIH research animal facility in standard vivarium cages with ad libitum food availability and 12-hour dark/light cycle. The experiments described here were conducted during light-period (typically between 9am and 7pm).

Wild animals	No wild animals were used in this study
Field-collected samples	No field-collected samples were used in this study
Ethics oversight	All experimental procedures were approved by the National Institute of Diabetes and Digestive and Kidney Diseases/NIH

Note that full information on the approval of the study protocol must also be provided in the manuscript.
