# Supplementary material for: Orbitofrontal-striatal potentiation underlies cocaine-induced hyperactivity
Source: Nat Commun. 2020 Aug 10;11:3996. doi: 10.1038/s41467-020-17763-8 (PMC7417999; doi:10.1038/s41467-020-17763-8)
Supplement: Supplementary file 2 — Peer Review File [file 41467_2020_17763_MOESM2_ESM.docx]

Peer Review Information

**Journal:** Nature Communications

**Manuscript title:** Orbitofrontal-striatal potentiation underlies cocaine-induced hyperactivity

**Corresponding author name(s):** Dr Alexxai Kravitz

**Editorial notes:**

**Transferred manuscripts**

This manuscript has been previously reviewed at another journal that is not operating a transparent peer review scheme. This document only contains reviewer comments and rebuttal letters for versions considered at *Nature Communications*.

**Reviewer comments & decisions:**

**Reviewer comments, first version:**

Reviewer #2 (Remarks to the Author):

This is a nice study that oulines changes in corticostriatal activity by stimulants in vivo.

We appreciate the attention to critiques and think this revised manuscript more appropriately frames the scope of the study, and more carefully deals with the treatment of inter- vs. intra-subject quantitative analysis. This response to reviewers potentially begins to deal with the question of dopamine receptor-specific signaling underlying the central findings of this study, but appears to be missing a critical supplemental figure supporting this point.

Major critique:

We remain confused about the design of the mixed-antagonist “Anti-DA” experiments. The response mentions a new experiment with decreased D1-antagonist concentration, but it appears the relevant supplemental figure is missing from the submitted manuscript (Supplemental Figure 4).

additional less major critques:

-There are some concerns raised by the Figure 2 photometry experiments. Nonspecific-looking (bidirectional) broadening in photometry traces after cocaine challenge suggest substantial movement artifacts that—even if they themselves do not rise to the author’s defined event threshold—may contribute to the rate of threshold-crossing events. As GcAMP kinetics are relatively slow, it would be possible to introduce an additional temporal criteria for events, depending on the temporal character of movement artifacts. At the least, it would be helpful to have a portion of the trace presented at a timescale at which individual events are visible to compare with previous recordings of these populations during locomotion.

-Also with respect to fiber photometry, how do absolute fluorescence changes compare between subjects? Is it reasonable to threshold events and aggregate data without normalizing dF/F magnitudes?

-While we can infer that the effects are condition-specific, a saline control in Figure 3K would still be proper, as suggested by reviewer 1.

-RE point #4 in original review, the major question is if the degree of activity change —either by aggregate single units/LFPs or by fiber photometry— correlate with locomotor phenotype/degree of sensitization? Once you calculate velocities of the animals, does this correlate with the population increase in either D1- or D2-SPNs? We think if the data is available, this is important to include.


Reviewer #3 (Remarks to the Author):

The authors have comprehensively addressed my prior comments. The manuscript is clear and statistical analyses are rigorous. The findings are an important contribution to the literature. I have no further concerns.


Here is my assessment of the authors' responses to reviewer 1:

The authors have not fully addressed Reviewer 1’s first concern, as the abstract and introduction are still initially framed (in the first sentence of each) in terms of examining the effects of prescribed stimulants and differentiating therapeutic versus adverse effects. These sentences could be revised to more narrowly focus on stimulant-induced hyperactivity. The authors have addressed all of Reviewer 1's other concerns satisfactorily.

**Author rebuttal, first version:**

Reviewer 2, Major critique:

We remain confused about the design of the mixed-antagonist “Anti-DA” experiments. The response mentions a new experiment with decreased D1-antagonist concentration, but it appears the relevant supplemental figure is missing from the submitted manuscript (Supplemental Figure 4).

We truly apologize for this omission. The reviewer correctly notes that Supplemental Figure 4 was inadvertently left out of the text when we re-formatted the document to put figures “in-line” with the text for ease of reviewing. In this resubmission we are including publication-ready figures, including Supplemental Figure 4.

Additional less major critques:

-There are some concerns raised by the Figure 2 photometry experiments. Nonspecific-looking (bidirectional) broadening in photometry traces after cocaine challenge suggest substantial movement artifacts that—even if they themselves do not rise to the author’s defined event threshold—may contribute to the rate of threshold-crossing events. As GcAMP kinetics are relatively slow, it would be possible to introduce an additional temporal criteria for events, depending on the temporal character of movement artifacts. At the least, it would be helpful to have a portion of the trace presented at a timescale at which individual events are visible to compare with previous recordings of these populations during locomotion.

We appreciate the reviewer concern over identifying movement artifacts as we believe this is a fundamental issue in the analysis of calcium photometry transients. Our approach includes photometry recordings from eYFP-expressing animals to identify calcium-insensitive changes in activity. While some labs have used the “isosbestic” point (405nm) GCaMP for this purpose, in our experience, and in conversations with others we believe this approach can incur serious flaws (see: https://twitter.com/CodySiciliano/status/1194710301733638145). For this reason, we recorded from separate animals that express GFP instead of GCaMP in this study.

In the interest of transparency, we reported the existence of small movement artifacts, which in our experience and discussions with peers are often present in eYFP animals. This is what motivated us to set a threshold to identify true calcium transients from potential movement artifacts. We determined that the vast majority of these artifacts in eYFP mice were below a 5% change in fluorescence, so we applied this threshold to GCaMP6s signals in D1-Cre and A2a-Cre mice as well. While we can appreciate the reviewer’s idea to improve our transient detection approach, striatal photometry population transients often summate, creating complex transient shapes. Therefore, adding a temporal criterion for peak decay might be misleading. Most importantly, however, we believe that as long as the same criteria is applied to both pathways and normalized within animal, they are fair. The main conclusion of this experiment was that these pathways did not exhibit *opposing* changes in activity, but were increased in both pathways. We believe that that is unlikely to be dependent on the specifics of how individual transients are identified. This said, we agree with the reviewer’s comment about showing a more informative portion of photometry traces in Figure 2, which we have now done. We now included example photometry traces at a much smaller time-scale for both dMSN and iMSN recordings (Figure 2d).

-Also with respect to fiber photometry, how do absolute fluorescence changes compare between subjects? Is it reasonable to threshold events and aggregate data without normalizing dF/F magnitudes?

We thank the reviewer for the comment and for the opportunity to clarify this important point. Briefly, we normalize the photometry traces as dF/F before performing any analyses. We have now highlighted this point in our methods.

We were guided in our approach by one of the first papers using calcium photometry as a read-out of neuronal activity in direct and indirect striatal pathways (Cui et al., 2013; now added as a reference), which also normalized all traces as changes in fluorescence (dF) over total fluorescence (F) prior to any analysis. We do not think it is meaningful to analyze raw fiber photometry fluorescence without this normalization. The raw fluorescence levels in individual mice are subject to slight variabilities in GCaMP virus expression and fiber micropositioning, which are both impossible for the experimenter to control accurately enough to analyze raw signals. Thus, we normalize all signals to dF/F before proceeding with any further analysis, including the thresholding of transient events. We have now highlighted this in the methods section of the revised manuscript.

-While we can infer that the effects are condition-specific, a saline control in Figure 3K would still be proper, as suggested by reviewer 1.

We agree with this point, but as mentioned in our prior rebuttal both eYFP and ChR2 groups escalated their responses to repeated injections of the same dose of cocaine (20mg/Kg) over several contiguous days, which is a behavioral readout of sensitization that does not normally occur in saline treated animals. However, in this revised discussion of the manuscript, we more fully discuss this limitation of this study (highlighted in yellow). Specifically, we acknowledge the need for a proper saline control to fully understand how our high-frequency stimulation affects cocaine-induced locomotor sensitization, and note that future experiments will be needed to fully address this aspect of the results. We believe that the data presented in this manuscript fully support our more limited conclusions on the changes in locomotion.

-RE point #4 in original review, the major question is if the degree of activity change —either by aggregate single units/LFPs or by fiber photometry— correlate with locomotor phenotype/degree of sensitization? Once you calculate velocities of the animals, does this correlate with the population increase in either D1- or D2-SPNs? We think if the data is available, this is important to include.
We thank the reviewer for this very interesting point. This data does exist, and we have analyzed it as the reviewer suggests (the analysis has been included in Supplementary figure 2d). All mice (n=6 iMSN, n=8 dMSN mice) increased velocity with cocaine, and 12/14 mice had an increase in calcium transient rate after cocaine. However, these two factors did not themselves correlate, demonstrating that the increase in calcium rate was not solely attributable to the increase in velocity. This is consistent with prior recordings in these populations using 2-photon microscopy (Parker et al., 2018) or head-mounted miniscopes (Barbera et al., 2016), which both show strong increases in activity of both pathways between rest (velocity <2cm/s) and movement (velocity >2cm/s), but do not show linear increases in activity in either pathway above ~2cm/s. We also previously reported a similar result with fiber photometry (Fobbs et al., 2019).

Finally, we believe that this analysis also argues against the increase in frequency of calcium events being an artifact due to increased movement speed upon cocaine administration, as noted in the above comment. If that was the case, we would expect to find a positive correlation between velocity and frequency of calcium events, which was not the case. We have now included this data as Supplementary figure 2d, as well as a description in the main text of the revised manuscript.

Reviewer #3 (Remarks to the Author):

The authors have comprehensively addressed my prior comments. The manuscript is clear and statistical analyses are rigorous. The findings are an important contribution to the literature. I have no further concerns.
We thank the reviewer for his/her appreciation of our efforts in improving the manuscript.

Here is my assessment of the authors' responses to reviewer 1:

The authors have not fully addressed Reviewer 1’s first concern, as the abstract and introduction are still initially framed (in the first sentence of each) in terms of examining the effects of prescribed stimulants and differentiating therapeutic versus adverse effects. These sentences could be revised to more narrowly focus on stimulant-induced hyperactivity. The authors have addressed all of Reviewer 1's other concerns satisfactorily.

We thank the reviewer for commenting on Reviewer #1’s concerns. In the revised version of the manuscript we have adjusted the text accordingly. In particular, we have now fully focused both the abstract and the introduction on the effects of psychostimulants on both animal’s behavior and cortico-striatal circuit mal-adaptations.

**Reviewer comments, second version:**

Reviewer #2 (Remarks to the Author):


We appreciate the authors' attention to our comments and the changes made. Specifically, we appreciate the effort put into inclusion of velocity data and the new correlational analysis. We hope to read a more targeted investigation of D1 vs D2-mediated effects in a future study.

**Author rebuttal, second version:**

**Reviewer comments, third version:**

**Author rebuttal, third version:**
